# Supplementary material for: The critical role of optimized energy density in controlling void morphology and enhancing mechanical properties of L-PBF Ti-6Al-4V ELI alloy
Source: PLoS One. 2025 Jun 3;20(6):e0325276. doi: 10.1371/journal.pone.0325276 (PMC12133182; doi:10.1371/journal.pone.0325276)
Supplement: Attachment — Submitted filename: Summary of Comments on PONE-D-24-50441_reviewer-reviewed.pdf [file pone.0325276.s001.pdf]

The description of the manufacture of the samples needs significant improvement, detailing all the samples manufactured, their sizes, number off etc for each of the tests performed in their analysis.

The testing methods need to be more detailed to provide details of the sampling methods used to provide statistical robustness. Also, any stated value must have an associated error value stated.

The xCT software should be utilised in the analysis to extract useful information regarding the pores, including e.g. pore volume, pore size distributions, pore geometries (average roundness, average axis dimensions), etc. There is a lot of valuable data possible from xCT that you are not using.

There appears to be discrepancy between the results in Fig 3 and those in Fig 4. This needs to be explained.

The discussion of the results is quite limited and needs improvement, with further analysis of the data required. Some conclusions and hypotheses are made that are unsubstantiated by the results as they stand and more detailed analysis of the results, and further sample analysis (advanced SEM techniques) will be required to be able to substantiate these.

PLOS ONE

The critical role of optimized energy density in controlling void morphology and enhancing mechanical properties of L-PBF Ti-6Al-4V ELI alloy

--Manuscript Draft--

**Manuscript Number:**	PONE-D-24-50441
**Article Type:**	Research Article
**Full Title:**	The critical role of optimized energy density in controlling void morphology and enhancing mechanical properties of L-PBF Ti-6Al-4V ELI alloy
**Short Title:**	Optimized energy density for controlling void morphology and mechanical properties of L-PBF Ti-6Al-4V alloy
**Corresponding Author:**	Viritpon Srimaneepong<br>Chulalongkorn University Faculty Of Dentistry<br>Bangkok, Bangkok THAILAND
**Keywords:**	Ti-6Al-4V ELI; Laser Powder Bed Fusion; Energy density; Laser power; mechanical properties; Void morphology
**Abstract:**	Laser power is referred to as one of the critical process parameters governing the volumetric energy density in the Laser Powder Bed Fusion (L-PBF) process. The purpose of the study is to systematically investigate the influence of laser energy density on the void morphology, microstructure, and mechanical properties of the L-PBF printed parts which were fabricated with laser power ranging from 75 to 175 W. Comprehensive analysis of void defect was conducted by employing Archimedes' method, optical microscope (OM), and X-ray microcomputed tomography (Micro-CT). Surface quality was analyzed by surface roughness measurement. Tensile testing was performed to establish the correlation between process parameters, material microstructure, and mechanical behavior in as-built samples. Under the optimal process parameters, this work achieved a minimum void fraction of 0.3 %. At various laser energy densities, three distinct morphologies, namely lack of fusion (LOF), gas pores (GP), and keyhole (KH), were generated. Notably, LOF has a more detrimental effect on tensile characteristics, in comparison to GP and KH defects if laser power was less than 100W. Interestingly, subsurface spherical pores at the hatch border demonstrate a less substantial influence on the tensile behavior of as-built samples than LOF. The correlation analysis revealed that the presence of void defects primarily influenced strength, modulus of elasticity, and strain at break. Energy density proved to play a pivotal role in defect generation, non-equilibrium microstructure, and mechanical properties of L-PBF. Based on our findings, selecting 100W of laser power with a speed of 1200 mm/sec could be an optimal choice for achieving a satisfactory result in as-built L-PBF part.
**Order of Authors:**	Supapat Trithepchunlayakoon
	Aung Nyein Soe
	Atikom Sombatmai
	Suppakrit Khrueaduangkham
	Vorapat Trachoo
	Dinesh Rokaya
	PATCHARAPIT PROMOPPATUM
	Viritpon Srimaneepong
**Additional Information:**	
**Question**	**Response**
**Financial Disclosure**<br><br>Enter a financial disclosure statement that	Yes

Powered by Editorial Manager® and ProduXion Manager® from Aries Systems Corporation

Summary of Comments on PONE-D-24-50441_reviewer-reviewed.pdf

This page contains no comments

describes the sources of funding for the work included in this submission. Review the submission guidelines for detailed requirements. View published research articles from *PLOS ONE* for specific examples.<br><br>This statement is required for submission and **will appear in the published article** if the submission is accepted. Please make sure it is accurate.<br><br>**Funded studies**<br>Enter a statement with the following details:<br>• Initials of the authors who received each award<br>• Grant numbers awarded to each author<br>• The full name of each funder<br>• URL of each funder website<br>• Did the sponsors or funders play any role in the study design, data collection and analysis, decision to publish, or preparation of the manuscript?<br><br>Did you receive funding for this work?	
Please add funding details.<br>as follow-up to "**Financial Disclosure**<br><br>Enter a financial disclosure statement that describes the sources of funding for the work included in this submission. Review the submission guidelines for detailed requirements. View published research articles from *PLOS ONE* for specific examples.<br><br>This statement is required for submission and **will appear in the published article** if the submission is accepted. Please make sure it is accurate.	The Faculty of Dentistry, Chulalongkorn University (grant no: DF65003) and the National Research Council of Thailand (NRCT) (grant no: N23A650883).

Powered by Editorial Manager® and ProduXion Manager® from Aries Systems Corporation

This page contains no comments

**Funded studies**<br>Enter a statement with the following details:<br>• Initials of the authors who received each award<br>• Grant numbers awarded to each author<br>• The full name of each funder<br>• URL of each funder website<br>• Did the sponsors or funders play any role in the study design, data collection and analysis, decision to publish, or preparation of the manuscript?<br><br>Did you receive funding for this work?"	
Please select the country of your main research funder (please select carefully as in some cases this is used in fee calculation).<br>as follow-up to "**Financial Disclosure**<br><br>Enter a financial disclosure statement that describes the sources of funding for the work included in this submission. Review the submission guidelines for detailed requirements. View published research articles from *PLOS ONE* for specific examples.<br><br>This statement is required for submission and **will appear in the published article** if the submission is accepted. Please make sure it is accurate.<br><br>**Funded studies**<br>Enter a statement with the following details:<br>• Initials of the authors who received each award<br>• Grant numbers awarded to each author<br>• The full name of each funder<br>• URL of each funder website<br>• Did the sponsors or funders play any role in the study design, data collection and analysis, decision to publish, or preparation of the manuscript?<br><br>Did you receive funding for this work?"	THAILAND - TH
**Competing Interests**	The authors have declared that no competing interests exist.

This page contains no comments

Use the instructions below to enter a competing interest statement for this submission. On behalf of all authors, disclose any competing interests that could be perceived to bias this work—acknowledging all financial support and any other relevant financial or non-financial competing interests.<br><br>This statement is required for submission and **will appear in the published article** if the submission is accepted. Please make sure it is accurate and that any funding sources listed in your Funding Information later in the submission form are also declared in your Financial Disclosure statement.<br><br>View published research articles from *PLOS ONE* for specific examples.<br><br>**NO authors have competing interests**<br><br>Enter: *The authors have declared that no competing interests exist.*<br><br>**Authors with competing interests**<br><br>Enter competing interest details beginning with this statement:<br><br>*I have read the journal's policy and the authors of this manuscript have the following competing interests: [insert competing interests here]*<br><br>* typeset	
**Ethics Statement**<br><br>Enter an ethics statement for this submission. This statement is required if the study involved:<br><br>• Human participants<br>• Human specimens or tissue<br>• Vertebrate animals or cephalopods<br>• Vertebrate embryos or tissues	N/A

Powered by Editorial Manager® and ProduXion Manager® from Aries Systems Corporation

This page contains no comments

• Field research<br><br>Write "N/A" if the submission does not require an ethics statement.<br><br>General guidance is provided below. Consult the submission guidelines for detailed instructions. **Make sure that all information entered here is included in the Methods section of the manuscript.**	

Powered by Editorial Manager® and ProduXion Manager® from Aries Systems Corporation

This page contains no comments

**Format for specific study types**<br><br>**Human Subject Research (involving human participants and/or tissue)**<br>• Give the name of the institutional review board or ethics committee that approved the study<br>• Include the approval number and/or a statement indicating approval of this research<br>• Indicate the form of consent obtained (written/oral) or the reason that consent was not obtained (e.g. the data were analyzed anonymously)<br><br>**Animal Research (involving vertebrate animals, embryos or tissues)**<br>• Provide the name of the Institutional Animal Care and Use Committee (IACUC) or other relevant ethics board that reviewed the study protocol, and indicate whether they approved this research or granted a formal waiver of ethical approval<br>• Include an approval number if one was obtained<br>• If the study involved *non-human primates*, add *additional details* about animal welfare and steps taken to ameliorate suffering<br>• If anesthesia, euthanasia, or any kind of animal sacrifice is part of the study, include briefly which substances and/or methods were applied<br><br>**Field Research**<br><br>Include the following details if this study involves the collection of plant, animal, or other materials from a natural setting:<br>• Field permit number<br>• Name of the institution or relevant body that granted permission	
**Data Availability**<br><br>Authors are required to make all data underlying the findings described fully available, without restriction, and from the time of publication. PLOS allows rare exceptions to address legal and ethical concerns. See the PLOS Data Policy and FAQ for detailed information.	No - some restrictions will apply

Powered by Editorial Manager® and ProduXion Manager® from Aries Systems Corporation

This page contains no comments

A Data Availability Statement describing where the data can be found is required at submission. Your answers to this question constitute the Data Availability Statement and **will be published in the article**, if accepted.<br><br>**Important:** Stating 'data available on request from the author' is not sufficient. If your data are only available upon request, select 'No' for the first question and explain your exceptional situation in the text box.<br><br>Do the authors confirm that all data underlying the findings described in their manuscript are fully available without restriction?	
**Describe where the data may be found in full sentences. If you are copying our sample text, replace any instances of XXX with the appropriate details.**<br><br>• If the data are **held or will be held in a public repository**, include URLs, accession numbers or DOIs. If this information will only be available after acceptance, indicate this by ticking the box below. For example: *All XXX files are available from the XXX database (accession number(s) XXX, XXX.*).<br>• If the data are all contained **within the manuscript and/or Supporting Information files**, enter the following: *All relevant data are within the manuscript and its Supporting Information files.*<br>• If neither of these applies but you are able to provide **details of access elsewhere**, with or without limitations, please do so. For example:<br><br>*Data cannot be shared publicly because of [XXX]. Data are available from the XXX Institutional Data Access / Ethics Committee (contact via XXX) for researchers who meet the criteria for access to confidential data.*<br><br>*The data underlying the results presented in the study are available from (include the name of the third party*	Data will be made available on request.

This page contains no comments

and contact information or URL).<br>• This text is appropriate if the data are owned by a third party and authors do not have permission to share the data.<br><br>* typeset	
Additional data availability information:	Tick here if your circumstances are not covered by the questions above and you need the journal’s help to make your data available.

This page contains no comments

Manuscript Click here to access/download;Manuscript;MS for PLOS one.pdf

The critical role of optimized energy density in controlling void morphology and enhancing mechanical properties of L-PBF Ti-6Al-4V ELI alloy

Supapat Trithepchunlayakoon[1], Aung Nyein Soe[2], Atikom Sombatmai[2], Suppakrit Khrueaduangkham[2], Vorapat Trachoo[3], Dinesh Rokaya[4,5], Patcharapit Promoppatum[2,**], Viritpon Srimaneepong[1,*]

[1] Department of Prosthodontics, Faculty of Dentistry, Chulalongkorn University, Henri Dunant road, Wang Mai, Pathum Wan, Bangkok, Thailand

[2] Department of Mechanical Engineering, Faculty of Engineering, King Mongkut's University of Technology Thonburi, Pracha Uthit road, Bang Mod, Thung Khru, Bangkok, Thailand

[3] Department of Oral surgery, Faculty of Dentistry, Chulalongkorn University, 34 Henri Dunant road, Wang Mai, Pathum Wan, Bangkok, Thailand

[4] Clinical Sciences Department, College of Dentistry, Ajman University, Ajman, United Arab Emirates.

[5] Center of Medical and Bio-Allied Health Sciences Research, Ajman University, Ajman, United Arab Emirates.

[*] Corresponding author
E-mail address: viritpon.s@chula.ac.th

[**] Co-corresponding author
E-mail address: patcharapit.pro@kmutt.ac.th

This page contains no comments

Abstract

Laser power is referred to as one of the critical process parameters governing the volumetric energy density in the Laser Powder Bed Fusion (L-PBF) process. The purpose of the study is to systematically investigate the influence of laser energy density on the void morphology, microstructure, and mechanical properties of the L-PBF printed parts which were fabricated with laser power ranging from 75 to 175 W. Comprehensive analysis of void defect was conducted by employing Archimedes' method, optical microscope (OM), and X-ray microcomputed tomography (Micro-CT). Surface quality was analyzed by surface roughness measurement. Tensile testing was performed to establish the correlation between process parameters, material microstructure, and mechanical behavior in as-built samples. Under the optimal process parameters, this work achieved a minimum void fraction of 0.3 %. At various laser energy densities, three distinct morphologies, namely lack of fusion (LOF), gas pores (GP), and keyhole (KH), were generated. Notably, LOF has a more detrimental effect on tensile characteristics, in comparison to GP and KH defects if laser power was less than 100W. Interestingly, subsurface spherical pores at the hatch border demonstrate a less substantial influence on the tensile behavior of as-built samples than LOF. The correlation analysis revealed that the presence of void defects primarily influenced strength, modulus of elasticity, and strain at break. Energy density proved to play a pivotal role in defect generation, non-equilibrium microstructure, and mechanical properties of L-PBF. Based on our findings, selecting 100W of laser power with a speed of 1200 mm/sec could be an optimal choice for achieving a satisfactory result in as-built L-PBF part.

This page contains no comments

Highlights

- The characteristics of void morphology were analyzed by three different methods.
- Different laser powers would generate different types of void defects.
- Irregular-shaped void defect has more detrimental effects in comparison to spherical ones.
- Inadequate or excessive laser power can contribute to a higher percentage of void fraction.
- Void defect significantly affects the mechanical properties of L-PBF parts, especially ductility.

Keywords:

Ti-6Al-4V ELI; Laser Powder Bed Fusion; Energy density; Laser power; Mechanical properties; Void morphology

Introduction

Laser Powder Bed Fusion (L-PBF) stands out as a notable additive manufacturing method that utilizes a high-intensity laser source to selectively melt and fuse metal powder layer-by-layer until the final shape is achieved on the metal substrate. This layer-wise process has been developed to fabricate complex and customized parts with minimal material waste, overcoming some of the limitations found in conventional techniques [1]. Due to their high mechanical properties and biocompatibility, titanium alloys, specifically Ti-6Al-4V, have been used for biomedical devices, including orthopedic prostheses, maxillofacial prostheses, and dental prostheses [2, 3].

This page contains no comments

However, producing high-quality Ti-6Al-4V components using L-PBF could be a challenge due to high-temperature gradients and a layer-wise approach, which are governed by various process parameters including laser power, scanning speed, layer thickness, and hatch spacing [4, 5]. To understand the effect of these parameters, volumetric energy density, a measurement of the averaged energy input applied to metal powder by the volume of melt pool formed during scanning illustrating the relationship between the key process parameters, has been extensively studied. This parameter critically determines melt pool characteristics, defect formation, surface roughness, and overall part quality [6, 7].

Among the challenges of L-PBF, the void defect is critical and sometimes inevitable, especially from an improper process parameter setting. Voids can manifest in various types including lack of fusion (LOF), gas pores (GP), and keyholes (KH). Each type has a distinct morphology related to different process parameters[8]. According to Kasperovich *et al.*, they investigated the porosity in 2D and 3D approaches and claimed that optimal process parameters can significantly reduce the void defect. They classified the void defect into two major types including defects from insufficient energy which are irregular-shape so-called lack of fusion defects and excessive energy which are near-spherical-shape called keyhole defects [9].

Although it is well known that L-PBF parts have a unique fine microstructure due to rapid melting and solidification, which makes L-PBF components possess superior mechanical strength compared to conventional techniques [10, 11], void defects play a role in the physical and mechanical properties of the as-built L-PBF work [12, 13]. Meng *et al.* noted that void defects significantly impact tensile strength, especially ductility. Moreover, the influence of defects can overcome the influence of microstructure when there is a high amount of void [12]. Gong *et al.* found that spherical voids might be less detrimental to mechanical properties than elongated ones; even a 1% lack of fusion can cause serious effects

This page contains no comments

on tensile properties because of the sharp border and crack-like shape that become the stress concentration, which weakens the component. In contrast, less than 5% of spherical voids have only a few effects on the part [13].

Besides void defects, another notable drawback of the L-PBF process is surface roughness owing to its layer-wise nature. Parts produced using L-PBF often exhibit a coarser surface compared to conventionally produced ones. This can affect mechanical properties, fatigue strength, wear resistance, and even biocompatibility in medical applications [14, 15]. Several factors contribute to this surface roughness, including the stairstep effect, scanning track contour and overlap, balling, spattering, and adhered powder [14-18]. Furthermore, Hassanin *et al.* highlighted the interplay between porosity and surface roughness and indicated that increased porosity can exacerbate surface roughness, particularly on the top surface [15].

Many researchers have attempted to optimize its parameters and focus on achieving complete melting to reduce the defects. However, previous literature offers inconsistent views on optimal parameters. Sun *et al.* noted that increasing laser power can improve tensile strength, but further increase beyond the optimum point had minimal effect on tensile strength [19]. Buhairi *et al.* also state that the variation in optimal laser power found in their literature review ranges from 70 W to 400 W [20]. Several researchers tried to create a process window to show the effect of process parameters on defect generation and use them to predict the type of void [8, 21, 22]. While Gong *et al.* found that porosity is not linearly related to energy density [8]. Some studies found that there is no single optimal energy density value that works for all L-PBF machines. Even the same value can result from various combinations of key parameters that cause different thermal behavior. Investigating the most suitable process parameter for each machine and application is necessary to produce a suitable workpiece and meet the standard requirements of biomedical applications.

This page contains no comments

Through a systematic experimental assessment, the current work was implemented to explore the impact of various laser energy densities on void defect generation, surface roughness, microstructure, and mechanical properties in the regime of lower laser energy inputs. Laser powers ranging from 75 to 175 W were chosen while keeping other process parameters constant. A comprehensive analysis of void defect would be carried out using Archimedes' method, optical micrographs, and micro-computed tomography. The results obtained from this study will be a fundamental understanding of correlations between defects and mechanical properties of as-built L-PBF Ti-6Al-4V alloy.

Materials and Methods

Materials and sample preparation

Ti-6Al-4V ELI alloy powder (AP&C GE Additive, Canada), with a particle size distribution of 15-45 µm and an apparent density of 2.49 g/cm^3 was used as powder feedstock. The test samples were additively fabricated using an L-PBF technique on a metal 3D printing machine (Truprint 1000, Trumpf Germany). To avoid contamination, the oxygen in the build chamber was purged using a high-purity shielding argon gas with a speed of 2.5 m/s. The oxygen level was kept below 0.1% during fabrication so that it would have a lesser effect on the qualities of as-built samples [23]. To investigate the characteristics influenced by laser energy inputs, different laser powers (75, 100, 125, 150 and 175 W) were chosen to obtain different volumetric energy densities with a fixed scanning speed of 1200 mm/sec, layer thickness of 20 µm, hatch spacing of 110 µm, laser spot size of 30 µm and Chessboard scanning pattern. The volumetric energy density (E_v) is expressed as follows in Eq. (1):

Number: 1 Author: Subject: Comment on Text Date: 18/12/2024 15:25:26

You need to provide a description of the manufacture of all the samples, i.e their geometries, numbers off, position on the bed relative to the recoater direction and gas flow direction (position on the bed can greatly influence quality due to weld spatter, obscuration of the laser by weld fumes, variation in bed density, variation in beam quality at extremes of bed etc).

How were the samples removed from the build plate?

$$E_v = \frac{P}{v \times h \times t} \quad (1)$$

where P is the laser power (W), v is the scanning speed (mm.s^{-1}), h is the hatch spacing (μm), and t is the layer thickness (μm). The volumetric energy density will be 28.41, 37.88, 47.35, 56.82, 66.29 J/mm^3 according to laser power.

Standard metallographic preparation was followed for sample characterization. The samples were sectioned along the build direction and hot-mounted in the epoxy resin. Then, the samples were ground with silicon carbide abrasive papers starting from No. 400 to 2000 on a mechanical polishing machine (Polishing Machine-Nano 2000, PACE Technologies, USA), followed by polishing with diamond suspension up to 1μm and colloidal silica suspension (Struers, OP-S). The samples were etched for 20 sec at room temperature with Kroll's reagent (2.5 mL hydrofluoric acid, 5 mL nitric acid, and 100 mL distilled water) to observe microstructures of as-built samples.

Investigation of surface roughness

In the present study, we investigated a surface roughness analysis for the top and side surfaces of each laser power group to assess the surface quality of samples using a non-contact profilometer (Alicona, infiniteFocus SL, Austria). The average surface roughness was reported as mean profile roughness (R_a), which is the integral of the absolute value of the surface profile that deviates from the mean line for a specific length, as given by Eq. (2).

$$Ra = \frac{1}{1}\int_0^1 |z(x)|\, dx \quad (2)$$

Number: 1 Author: Subject: Replacement Text Date: 18/12/2024 15:24:41
used was

Number: 2 Author: Subject: Cross-out Date: 18/12/2024 15:24:37
, we investigated a

Number: 3 Author: Subject: Inserted Text Date: 18/12/2024 15:24:33
 was investigated

Number: 4 Author: Subject: Comment on Text Date: 18/12/2024 15:24:29
How many samples were assessed? How many readings were made per sample?
What sample area was used?

where L is the evaluation length, $|z(x)|$ is the absolute value of the shaded area for the evaluation length, and dx is the surface profile segment.

Oxygen, Nitrogen, and Hydrogen content analysis

Since chemical species can affect the part integrity for medical applications, their contents in as-built samples need to be analyzed. Therefore, the O/N/H analyzer (EMGA-830, Horiba, Japan) was used to determine the concentration of oxygen, nitrogen, and hydrogen in all samples with different laser powers. Two non-dispersive infrared detectors measure oxygen as carbon monoxide and carbon dioxide during the analysis, while a thermal conductivity detector measures nitrogen, and a non-dispersive infrared detector measure hydrogen as H_2O.

Void defect analysis

Despite some limitations, Archimedes' method, Optical microscopy (OM), and X-ray micro-computed tomography (Micro-CT) methods are three widely used approaches for accessing void defects of L-PBF samples [24]. In the current study, we explored a combined utilization of these methods for the quantification of the void fraction and comprehensive analysis of void morphology and void distribution.

Following Archimedes' method, the samples were weighed in air and water to calculate void fraction (ϕ_{Arch}) (Eq.(3)) using an analytical balance (METTLER TOLEDO, classic, AB104-S, Switzerland) [25]. The measurement was repeated five times for each laser group. Although Archimedes' method is easy to implement, it is observed to have limitations that affect the precision of the result, especially when the defect contains unmelt powders [13].

Number: 1 Author: Subject: Comment on Text Date: 18/12/2024 15:24:25
What sample geometries, volumes, masses were used? How were the samples extracted from the L-PBF samples? How many samples were tested for each of the energy level groups?

Number: 2 Author: Subject: Cross-out Date: 18/12/2024 15:23:58
, we explored

Number: 3 Author: Subject: Inserted Text Date: 18/12/2024 15:23:53
were explored.

Number: 4 Author: Subject: Comment on Text Date: 18/12/2024 15:23:50
What was sample geometry, size?

Number: 5 Author: Subject: Comment on Text Date: 18/12/2024 15:23:45
Is that 5 different specimens for each energy group or one sample measured 5 times?

$$\phi_{Arch}(\%)=\frac{\rho_{bulk}-\rho_{sample}}{\rho_{bulk}}\times 100 = 1-\left(\frac{m_a}{m_a - m_{water}}\right)\frac{\rho_{water}}{\rho_{bulk}}\times 100 \quad (3)$$

Where ρ_{sample} is the density of the sample, m_a is the mass in air, m_{water} is the mass in water, is ρ_{water} the density of the water at 25 °C (ρ_{water} = 0.997 g/cm^3), and ρ_{bulk} is the bulk density of Ti-6Al-4V (ρ_{bulk} = 4.42 g/cm^3).

For the OM method, void fraction analysis was performed using five different optical micrographs taken from the polished cross-section of each group along the build orientation. Image analysis software (Image J) was used to convert a grayscale image into a binary image to separate voids from bulk metal, and a median filter with a radius of 2 pixels was applied to remove noises and contaminations originating from sample preparation. A void fraction (ϕ_{OM}) was calculated by dividing the area of black pixels by the total number of pixels in binary images (Eq. (4)). The advantage of this technique is that it reduces the amount of liquid trapped in the void defect. However, the void fraction obtained from this method is an estimated value that depends on how well the image represents the distribution of the defect [26].

$$\phi_{OM}(\%)=\frac{A_{void}}{A_{total}}\times 100 \quad (4)$$

Where A_{void} is the area of black pixels, and A_{total} is the area of the total pixels in binary images.

In addition to Archimedes' and OM methods, the In-vitro Micro-Computed Tomography (Skyscan 1173, BRUKER company, Belgium) with voxel size 6.05 µm was used to analyze the void defect in as-built samples with various laser powers. The three-dimensional model was then reconstructed by stitching together multiple two-dimensional

Number: 1 Author: Subject: Comment on Text Date: 18/12/2024 15:23:42
Need to have reference for all these values.

Number: 2 Author: Subject: Comment on Text Date: 18/12/2024 15:23:39
Is that 5 sections taken from one sample or 5 samples, each with one cross-section?
Sample size and geometry?

images that were projected during analysis from multiple slices of as-built samples. $\phi_{Micro\text{-}CT}$ is determined from the volume of void voxels divided by the total voxel of 3D reconstructed domains (Eq.(5)). However, caution should be taken since the final result of Micro-CT is dependent on the voxel size used in the analysis [24].

$$\phi_{Micro\text{-}CT}(\%)=\frac{V_{void}}{V_{total}}\times 100 \quad (5)$$

Where V_{void} is the volume of void voxels, and V_{total} is the total volume of voxels in the Micro-CT sample.

Mechanical testing

Following the ASTM E8, the tensile test was performed using a servo-hydraulic testing machine (Zwick Roell, HB 250, Germany) with a crosshead speed of 0.15 mm/min under quasi-static loading conditions, which is to keep a maximum strain rate of 0.001/S. The test was carried out in the same orientation as the building orientation and the displacement during loading was measured using a video extensometer attached to the machine. The ultimate tensile strength (σ_{UTS}), 0.2% offset yield strength (σ_y), Young's modulus (E), and the strain at break (ε) obtained from engineering stress-strain curves were also compared against the void fraction for each laser power group to understand the influence of laser energy density.

Microstructure and fractography

An optical microscope (OM, ZEISS Axio Imager 2, Germany) was used to investigate the void morphology and morphology of prior β grain of as-built Ti-6Al-4V ELI printed parts while a scanning electron microscope (SEM, FEI Quanta 250, USA) was used to characterize

Number: 1 Author: Subject: Comment on Text Date: 18/12/2024 15:23:36

This needs to be expanded on to determine the resolution of the system, e.g the minimum void diameter that can be resolved.

What is sample geometry and volume?
How many xCT taken of each energy level?

Number: 2 Author: Subject: Comment on Text Date: 18/12/2024 15:23:30

What was the sample geometry? Was this adhering to a standard, and if so, which one?

detailed features of non-equilibrium α' martensite that stemmed from the rapid heating and cooling rate during the L-PBF process. The same sample was used for OM and SEM analysis. Failure of as-built samples during tensile testing can be correlated to many factors including surface roughness, void defects, and non-equilibrium microstructure. To understand different modes of failure, we further investigated the fracture surfaces of broken tensile samples using SEM for each laser group.

Results and discussion

Investigation of surface roughness

The surface morphology of as-built samples at various laser powers is presented in Fig 1a. The result displays that the top surface of samples gets smoother with higher laser power due to the overlapping of scan tracks. This characteristic is supported by the result obtained from the measurement of mean profile roughness (Ra) on the top surface, as shown in Fig 1b. At 75 W, the top surface roughness is approximately 12.7 µm which is statistically significant than other groups. While 175 W reportedly achieved around 6 µm, which was 50 % lower than the former. The morphology of the side surface (Fig 1c), on the other hand, differs greatly from the top surface because it dominantly consists of adhered powders. Furthermore, there is no statistically significant difference in surface roughness between all laser groups, apart from 75 W and 175 W, which have slightly higher mean roughness values of 7.63 and 7.57 µm, respectively.

Fig 1. (a) 3D reconstructed surface topography of all samples from the software, (b) Mean surface roughness of the top surface, and (c) Mean surface roughness of the side surface.

Number: 1 Author: Subject: Comment on Text Date: 18/12/2024 15:23:25

Just one sample?

Number: 2 Author: Subject: Comment on Text Date: 18/12/2024 15:23:22

Need to include error value on all results.

The present finding demonstrates that the top surface roughness decreases with increasing laser power, which is a direct consequence of melt pool stability, greater overlapping of melting tracks, and fewer satellite particles (Fig 1a) and is consistent with Wang's study [27]. In contrast, the side surface roughness is a mix of the stair-step effect and powder-adhered roughness. This kind of roughness is often induced by powders along the lateral border of the sample that melt or partly melt because of heat radiation from the melt pool [28-30]. This study shows that increasing the laser from 75 W can reduce the surface roughness. The result corresponds with Molinari *et al.*'s work which states that increasing energy density reduces surface roughness because of the more continuous track and less adhered powder and balling effect [17]. However, a slight increase in surface roughness was observed in 175 W. The high energy input which generates a higher melt pool temperature can cause more powder to adhere to the vertical side of the samples [28]. Moreover, Wang noted that excessive energy density can also cause over-burning and outer edge bulge phenomena, which raise the surface roughness [18].

Oxygen, Nitrogen, and Hydrogen content analysis

In Table 1, the oxygen, nitrogen, and hydrogen contents in weight percentage (wt. %) of the as-built samples at different laser power groups are shown and compared with the Ti-6Al-4V ELI powder and standard ASTM data (ASTM F2924-14). The findings show that the O, N, and H contents of the as-built samples and Ti-6Al-4V ELI powder differ slightly. The O/N/H analysis further confirms the fact that, despite slightly higher O (wt.%) and N (wt.%), compositions in the as-built samples in this study did not exceed the ASTM standard.

Number: 1 Author: Subject: Comment on Text Date: 18/12/2024 15:23:19

Could also be due to a higher melt pool temperature and lower melt viscosity that affects the melt pool shape and therefore the surface quality. This should be researched and discussed in reference to the literature.

Table 1. The chemical composition from O/N/H analysis (wt.%)

Remark	O	N	H
Ti-6Al-4V powder	0.1012	0.0234	0.0033
75 W	0.1132	0.0274	0.0034
100 W	0.1124	0.0316	0.0032
125 W	0.1294	0.0308	0.0034
150 W	0.1189	0.0298	0.0031
175 W	0.1229	0.0322	0.0047
ASTM F2924-14	0.2 (Max)	0.05 (Max)	0.015(Max)

Void defect analysis

Fig 2 shows optical micrographs taken on cross-sections of as-built L-PBF samples with different laser powers. The 75 W group exhibits pores with irregular morphologies, which are commonly referred to as lack of fusion (LOF) and are likely caused by incomplete melting of powders in the melt pool during the L-PBF process. Whereas, in the samples fabricated at 100 and 125 W groups, small spherical gas pores (GP) are predominantly found. In contrast, the 150 and 175 W groups exhibit slightly different pore characteristics compared to those in the 100 and 125 W groups. The pore geometry appears to transition from round and semicircular to deep and narrow keyhole (KH) configuration, indicating deep penetration of the melt pool within alternative

Fig 2. Optical micrographs of as-built samples at various laser powers. (The same images were also used to calculate void fraction for the OM method.) layers.

Number: 1 Author: Subject: Comment on Text Date: 18/12/2024 15:23:17

These values need an error value after each one. You say that you measured 5 for each sample so your error should be:

standard deviation / sqareroot (5)

The micro-CT analysis was extended to gain insight into the formation of void defects in three-dimensional space inside the as-built samples. A cuboid of 1.5 × 1.5 × 1.5 mm^3 was extracted to display 3D void distribution and shape from all samples, including high magnification of void morphologies as shown in Fig 3. Although the presence of voids is relatively low at the laser power of 100 and 125 W, the number of voids increases when energy inputs of 75, 150, and 175 W are applied. The high magnification of pore morphology presented in Fig 3 is reasonably compatible with the observation previously stated in the OM analysis. Unlike the OM method, the result obtained from the Micro-CT approach can depict that the mode change from GP to KH begins at 125 W and becomes more noticeable at 150 and 175 W.

Fig 3. Cuboid of 3D geometry reconstructed by stitching 2D images from Micro-CT analysis. Higher magnification of void morphologies was also included.

According to Fig 2 and Fig 3, as a low energy input, 75 W, is assumed to be the main underlying reason behind the formation of numerous defects with elongated and irregular morphology. Insufficient energy input not only creates unstable melt pools but also causes poor overlapping between melting tracks and alternative layers, which in turn leads to entrapment of unmelt powders [31]. As the laser power continues to increase, this type of defect disappears and transitions to spherical gas pore (GP). GP may be formed by entrapped gas from within powders in the melt pool, which is unable to escape upon a high solidification rate [9]. On the other hand, KH mode, where the depth is greater than the width of the melt pool, is predominantly observed when laser power above 125 W is applied. According to a previous study, KH defect typically occurs when higher energy deposited on powder beds causes localized vaporization of metal powders that leads to the formation of a depression zone in the surface [32].

Number: 1 Author: Subject: Comment on Text Date: 18/12/2024 15:23:07

You should use the xCT software to extract useful information regarding the pores, including e.g pore size distributions, pore geometries (average roundness, average axis dimensions), etc. There is a lot of valuable data possible from xCT that you are not using.

Number: 2 Author: Subject: Comment on Text Date: 18/12/2024 15:23:11

This detail should be in the methodology section, including the methods used to extract the cubes.

Fig 4. (a) Comparison of void fraction among Archimedes' method, OM, and Micro-CT approach, (b) Comparison of void size distribution against various laser energy inputs, (c) Comparison of area void fraction along the sample thickness, and (d) Top view of a 3D reconstructed domain from micro-CT at the middle region.

In the current study, the comparison of void fractions using Archimedes' method, OM, and Micro-CT is shown in Fig 4a. All methods show a similar trend of the void fraction. The maximum void fraction was observed at 75 W, and as laser power increases, the void fraction decreases first before increasing after laser power exceeds 125 W. It showed that different energy inputs led to the fluctuation of void fraction in as-built L-PBF samples. This similar phenomenon has been previously described in the literature [11, 19, 27, 33-35].

It is also observed that at 75 W, OM yields a relatively higher void fraction compared to both Micro-CT and Archimedes' methods. For energy inputs other than 75 W, the void fraction obtained through OM and Micro-CT approaches are relatively comparable. Particularly at 150 W, the result depicts a high degree of similarity across different techniques. This observation aligns with prior findings [36], which also highlighted the tendency of the OM method to overestimate void fractions. The Micro-CT method, on the other hand, is found to underestimate the void fraction of LOF by around 30 % and 20% when compared to OM and Archimedes' methods, respectively. This could be attributed to the limitation of voxel size in this study, which fail to detect the void sizes lower than 18 µm.

Fig 4b depicts the void size distribution corresponding to different laser powers. Despite having different types with morphologies, the average 3D void sizes from Micro-CT are relatively consistent across all samples. It also showed that the average size of KH defect tends to increase slightly with higher laser power (Fig 4b), which is consistent with the study by du Plessis A [37].

Number: 1 Author: Subject: Comment on Text Date: 18/12/2024 15:22:55

What does this mean? Why do the images in Figure 4 not represent what is seen in Figure 3? Why is the density of pores so much higher in Figure 4? If a slice is taken near the centre of the images of those in Fig 3, the density of pores is much lower than shown in Fig 4 (unless Fig 4 is a cumulative image of many different layers?) Please explain this descrepancy, and provide more detail as to how Fig 4 is obtained.

Number: 2 Author: Subject: Comment on Text Date: 18/12/2024 15:23:05

What does this show? What are the black lines, are these error bars or the minimum and maximum levels? This is not clear.

A better method to show this data is using box-plots:

https://en.wikipedia.org/wiki/Box_plot

Number: 3 Author: Subject: Comment on Text Date: 18/12/2024 15:23:00

A useful metric would also be the circularity (or average axis lengths) as gas and lack of bonding pores are circular whilst KH pores are not, and this metric (from xCT) would be able to identify the distribution of each. Provide this information with accompanying data and discussion.

Additionally, we assessed void intensity throughout the thickness to highlight the variation in void fraction within each cross-section. Fig 4c presents the variation in area void fraction per slice. Apart from 75 W, all other groups exhibit a symmetrical void distribution pattern, characterized by higher intensity near the subsurface and lower intensity toward the center. In contrast, the 75 W group displays a unique void distribution pattern, featuring high-intensity peaks not only at the subsurface but also at 0.2 mm from the first peaks on both sides. Fig 4c also demonstrates that the peak intensity at the subsurface increases with higher laser power. Followingly, Fig 4d shows a top view of the 3D reconstructed void geometry in the middle region projected on the XZ plane for 75, 100, and 175 W. It becomes evident that the high-intensity peak recorded in Fig 4c originates from a greater number of roundish voids near the end of the hatch line within the hatch region. Particularly, the morphology of voids near the surface of as-built samples appears to be consistent across all laser power groups.

Martin *et al.* [38] suggested that the formation of subsurface pores in L-PBF samples is influenced by several factors such as insufficient overlapping between hatch tracks and contour tracks, laser power slowing down at the end of hatch tracks, and the laser momentarily switching off and jumping to neighboring tracks. In the current study, it could be due to the latter that deposits small spherical pores as the laser turns around at the end of the tracks. Therefore, it is found that the intensity of the peak at the border of the hatch region becomes higher with increasing laser power above 75 W due to localized metal vaporization caused by higher energy density [38, 39]. The presence of such subsurface pores is observed to affect the mechanical properties of L-PBF parts [40].

Number: 1 Author: Subject: Comment on Text Date: 18/12/2024 15:22:51
What is the void area? Is it the blue or the white? Please clarify on Figure.

Number: 2 Author: Subject: Replacement Text Date: 18/12/2024 15:22:40
is suggested

Number: 3 Author: Subject: Inserted Text Date: 18/12/2024 15:22:44
(

Number: 4 Author: Subject: Inserted Text Date: 18/12/2024 15:22:30
outer

Number: 5 Author: Subject: Replacement Text Date: 18/12/2024 15:22:48
postulated

Number: 6 Author: Subject: Comment on Text Date: 18/12/2024 15:22:26
You need to provide an explanation for the increased internal void peaks (second subsurface). I would suggest that it is due to power ramping of the laser that drops the powder below that required for complete fusion, before it rises up to the 75W. You need to determine from the system what the power ramping sequence used is, and how long would you expect the power to reach 75W from 0W (and thus, at a scan speed of 1200 mm/s, does this relate to the general width of the 2nd peaks)?

Alternatively, it could also be due to the scanner acceleration being low, so that the laser is at 75W whilst the scan speed is still well below 1200mm/s, increasing the energy density to values above which vaporisation and KH voids form.

You will need to investigate the shape of the pores in this peak to see if their morphology aligns with lack of fusion or key hole porosity.

Please include this and make the necessary discussion. Without this level of detail the discussion and results are fairly inconsequential as you have not identified the cause.

Mechanical testing

The engineering stress-strain curve obtained from tensile testing is exhibited in Fig 5a. which is observed to possess good reportability. There are statistically significant differences in yield stress, ultimate tensile stress, Young's modulus, and elongation at break among various laser powers (Fig 5b-e). According to Fig 5b and c, the lowest value of yield stress (820 MPa) and ultimate tensile stress (971 MPa) are obtained at 75 W, while the value tends to increase with increasing laser power. Unlike Young's modulus, the value increases as the laser power increases, however, above 75 W the value remains within the same range (Fig 5d). In the case of strain at break (Fig 5e), the value tends to rise with increasing laser power up to 100 W and subsequently drops. The 100 W group has the highest strain at break, which is approximately 6.5 %, followed by 125, 150, 175, and 75 W.

Fig 5. (a) Engineering stress and strain curve, (b) Yield stress (σ_y), (c) Ultimate tensile stress (σ_{UTS}), (d) Young's modulus (E), and (e) Strain at break (ε)

Through our analysis, we found that as-built L-PBF samples, apart from the 75 W, consistently showed superior yield stress and ultimate tensile stress, at ~1077 and ~1214 MPa, respectively, compared to wrought Ti-6Al-4V ELI alloy from previous literature [41, 42], which had less than 1000 MPa. In contrast, the strain at break of the current work was lower (less than 7%) which is lower than the wrought alloy of those mentioned works, which can rise greater than 10%. This is attributed to the characteristics of the L-PBF Ti-6Al-4V samples, which are characterized by high strength and low ductility. This behavior is primarily due to the formation of defects, fine acicular α′ martensitic microstructure, and residual stress [11, 34, 43, 44].

Number: 1 Author: Subject: Replacement Text Date: 18/12/2024 15:22:23
peatibility

Number: 2 Author: Subject: Comment on Text Date: 18/12/2024 15:22:19
All stated numbers must be quoted with error value.

Number: 3 Author: Subject: Replacement Text Date: 18/12/2024 15:22:14
and their

Number: 4 Author: Subject: Inserted Text Date: 18/12/2024 15:22:09
s

Number: 5 Author: Subject: Cross-out Date: 18/12/2024 15:22:06
s

Number: 6 Author: Subject: Cross-out Date: 18/12/2024 15:21:58
remains within the same range

Number: 7 Author: Subject: Replacement Text Date: 18/12/2024 15:22:02
, although

Number: 8 Author: Subject: Inserted Text Date: 18/12/2024 15:21:54
s are statistically equivalent.

Number: 9 Author: Subject: Replacement Text Date: 18/12/2024 15:21:51
increases from A.A% at 75W to 6.5% at 100W. After this, the value decreases with increasing laser power, e.g. B.B% (125W), C.C% (150W), D.D (175W).

Number: 10 Author: Subject: Comment on Text Date: 18/12/2024 15:21:46
Use the average of the values from 100-175W (with an error level).

Number: 11 Author: Subject: Comment on Text Date: 18/12/2024 15:21:43
Use the average of the values from 100-175W (with an error level).

Number: 12 Author: Subject: Comment on Text Date: 18/12/2024 15:21:35
Need values for yield and UTS and provide those values with references.

Number: 13 Author: Subject: Cross-out Date: 18/12/2024 15:21:24
of those mentioned works

Number: 14 Author: Subject: Inserted Text Date: 18/12/2024 15:21:31
for all energies,

Number: 15 Author: Subject: Inserted Text Date: 18/12/2024 15:21:27
that of

Number: 16 Author: Subject: Comment on Text Date: 18/12/2024 15:21:17
Need to provide the specific reference for the value, not two references.

Number: 17 Author: Subject: Inserted Text Date: 18/12/2024 15:21:20
[REF]

Number: 18 Author: Subject: Replacement Text Date: 18/12/2024 15:21:14
has been previously associated with

Number: 19 Author: Subject: Comment on Text Date: 18/12/2024 15:21:10
You need to provide a specific reference to each of the points raised, e.g. one for defects, one for microstructure and one for stress.

Fig 6. Correlation between tensile properties and the void fraction from Archimedes' method. (a) Yield stress (σ_y), (b) Ultimate tensile stress (σ_{UTS}), (c) Young's modulus (E), and (d) Strain at break (ε)

In addition, the tensile properties against ϕ_{Arch} for each laser power is displayed in Fig 6. All values of tensile properties decrease as ϕ_{Arch} get increased, demonstrating that the presence of voids has a major effect on the tensile properties of as-built samples, especially the 75 W group which has the highest void fraction.

The void fraction was found to play a substantial role in determining mechanical properties, especially strain at break, with inverse correlation for all modes (i.e., LOF, GP, and KH) as depicted in Fig 5e. In contrast, the tensile strength appears to be primarily influenced by α' grain refinement induced by increasing laser power above 75 W [45]. In general, the correlation analysis implemented in this study suggests that the microstructure has a smaller effect on mechanical characteristics than internal defects in L-PBF work, which is consistent with previous studies [13, 27].

As previously stated, variations in laser energy inputs resulted in various modes of defects. From the tensile results, the LOF in the 75 W has a particularly detrimental effect. The irregular morphology of LOF can act as stress concentration points, initiating cracks and leading to premature fracture under tensile loading (Fig 5a). This observation is consistent with prior research highlighting the significant impact of even a small amount of LOF on the mechanical properties of L-PBF parts [9, 13].

Microstructure and fractography

The microstructure characterization on an as-built sample was implemented for each laser group. Although Ti-6Al-4V is typically a dual phase (α + β) alloy, it is obvious that the

Number: 1 Author: Subject: Comment on Text Date: 18/12/2024 15:21:04
Why use this measurement of density? State why you are using this and not the other methods used.

Number: 2 Author: Subject: Comment on Text Date: 18/12/2024 15:20:58
I would like to see the points for each of the five energy levels displayed differently (different colours or different icons) on each graph so that the link between the energy level density and mechanical property can be seen easily in one graph.

Number: 3 Author: Subject: Comment on Text Date: 18/12/2024 15:20:55
has previously been shown to be

Number: 4 Author: Subject: Comment on Text Date: 18/12/2024 15:20:51
This statement is not justified since you have no data on α' grain size refinement for these samples. You will need to provide microstructural analysis, with grain size distributions for the α' martensite grains to be able to state this. Please do so.

The images in Fig 7 do are not suitable for this analysis and do not show anything mre than a typical Ti64 microstructure. They do not add value to the argument or discussion.

Number: 5 Author: Subject: Comment on Text Date: 18/12/2024 15:20:48
You need to extend the analysis of this microstructure to show whether or not any α' Martensite grain refinement is occurring.

microstructure of as-built samples is predominantly α' martensitic structure regardless of laser energy input, as displayed in Fig 7. The microstructures are characterized by acicular features of α' martensite with varying grain sizes within the prior β grains. In addition, the orientations of α' grains in the microstructure presumably vary with the size of the major axis. The grains with similar sizes tend to be parallel to one another, but smaller grains indicate a tendency to orient at 45° to the bigger ones.

Fig 7. Scanning electron microscopy (SEM) images of as-built samples.

The fracture morphology of all samples was analyzed to gain insight into the correlation between the manufacturing process and properties. Fig 8 shows the mode of failure for the respective laser groups. Following Fig 8, the middle region of the sample in each group exhibited a fibrous fracture, which is likely a ductile fracture, but the periphery of the samples displayed a thin shear lip at 45° along the tensile loading direction. However, each group's core area has unique features, and the width of the share lip is observed to vary in response to laser energy inputs. For 75 W, the central area has many irregular pores, uncombined regions, and unmelt powders. Additionally, the fracture surface of 75 W shows a relatively thin shear lip in comparison to other groups, indicating that the samples prematurely failed during tensile testing. Meanwhile, mixed modes of micro-dimples and cleavage fractures including a few spherical pores are typically found in the middle region of 175 W. In contrast to 75 W and 175 W, the middle area of 100 W is mainly dominated by the presence of a greater number of dimple networks and a few cleavage fractures. Moreover, the shear lip is substantially broader compared to the former, reflecting the necking behavior of the samples before tensile failure.

Fig 8. Fracture surface of as-built tensile samples from 75 W, 100 W, and 175 W groups. Higher magnification of specific areas was selected to represent the fracture behavior.

This page contains no comments

As illustrated in Fig 8, the fracture surface of all laser powers starting from 75 to 175 W exhibits different features in the periphery and middle region. For 75W, it is evident that the uncombined region with unmelt particles can degrade the load-bearing capacity of the cross-section during tensile loading. Additionally, the presence of small subsurface pores along the hatch region was observed, but their impact on the tensile properties of as-built L-PBF samples appeared to be less significant compared to LOF. This is typically due to their lower subsurface pore fraction at 75 W (~3.8 %) compared to 175 W (~12.5 %) (Fig 4c). Notably, in the 75 W, the width of the shear lip in the periphery region closely matched the distance between the subsurface pores region and pores intensity peak at the middle region where LOF was prevalent (Fig 4c and d), a phenomenon supported by Hu *et al.* [40]. In contrast, for other laser power groups, the ductile fracture in the central area was typically induced by the formation of the micro-voids during tensile loading. These observations underscore the critical influence of pores, particularly their void morphology, on the fracture behavior of the L-PBF samples. Furthermore, numerous micro-dimples or dimple networks were observed on the fracture surface of the same as-built samples produced at all laser power, which is an indication of plastic deformation [46, 47].

Conclusions

In the present study, an investigation was carried out to assess the impact of energy density on the formation of void defects, surface quality, microstructure characteristics, and mechanical properties. Key findings include:

- Selecting optimal parameters for L-PBF machines is key to minimizing defects and maximizing mechanical strength in parts, though challenges persist even with optimal settings.

This page contains no comments

- Surface roughness measurements revealed that as energy density increased, top surface roughness in as-built samples decreased due to better overlap of melting tracks, while side surface roughness stayed relatively unchanged, consisting mostly of partially adhered powder.
- Energy density variations led to three specific void defects: lack of fusion (LOF) at low energy, gas pores (GP) at higher energy, and keyhole (KH) defects at excessive energy.
- All three methods: Archimedes' method, optical microscopy (OM), and micro-computed tomography (Micro-CT) exhibited a similar trend of void fraction, however, Micro-CT specifically showed a variation in area void fraction across the sample thickness and revealed sub-surface pores.
- Tensile tests on as-built samples indicated that LOF was a major cause of premature fracture at 75 W under mechanical stress. In samples with prevalent GP and KH, strain at break was highly sensitive to void fraction, whereas tensile strength was influenced by α' grain refinement. Notably, subsurface pores impacted tensile strength less than internal pores.

Credit authorship contributions

S. Trithepchunlayakoon: Investigation, Formal analysis, Data curation, Writing-original draft, **A.N. Soe**: Formal analysis, Data curation, Writing-review & editing, **A. Sombatmai**: Validation, **S. Khrueaduangkham**: Visualization, **V. Trachoo**: Visualization, **D. Rokaya**: Validation, **P. Promoppatum**: Conceptualization, Methodology, Writing-review & editing, Project administration, **V. Srimaneepong**: Conceptualization, Methodology, Writing-review & editing, Funding acquisition

Number: 1 Author: Subject: Comment on Text Date: 18/12/2024 15:20:42
This conclusion cannot yet be substantiated by the results.

Acknowledgements

This work was supported by the Faculty of Dentistry, Chulalongkorn University (grant no: DF65003) and the National Research Council of Thailand (NRCT) (grant no: N23A650883). The authors appreciate the support from Dr. Ammarueda Issariyapat and Horiba (Thailand) for ONH analysis. Aung Nyein Soe acknowledges the financial support from King Mongkut's University of Technology Thonburi under the industrial Post-Doctoral and Post-Master program.

Data availability

Data will be made available on request.

Declaration of Generative AI and AI-assisted technologies in the writing process

During the preparation of this work, the authors used ChatGPT to improve readability and language. After using this tool, the authors reviewed and edited the content as needed and took full responsibility for the content of the publication.

Declaration of Competing Interest

The authors declare no conflict of interest.

This page contains no comments

References

1. Vandenbroucke B, Kruth JP. Selective laser melting of biocompatible metals for rapid manufacturing of medical parts. Rapid Prototyping Journal. 2007;13(4):196-203.

2. Khorasani A, Goldberg M, Doeven E, Littlefair G. Titanium in Biomedical Applications—Properties and Fabrication: A Review. Journal of Biomaterials and Tissue Engineering. 2015;5:593-619.

3. Jorge JRP, Barão VA, Delben JA, Faverani LP, Queiroz TP, Assunção WG. Titanium in dentistry: historical development, state of the art and future perspectives. J Indian Prosthodont Soc. 2013;13(2):71-7.

4. Yap CY, Chua CK, Dong ZL, Liu ZH, Zhang DQ, Loh LE, et al. Review of selective laser melting: Materials and applications. Applied Physics Reviews. 2015;2(4):041101.

5. Hanzl P, Zetek M, Bakša T, Kroupa T. The Influence of Processing Parameters on the Mechanical Properties of SLM Parts. Procedia Engineering. 2015;100:1405-13.

6. Khorasani A, Gibson I, Awan US, Ghaderi A. The effect of SLM process parameters on density, hardness, tensile strength and surface quality of Ti-6Al-4V. Additive Manufacturing. 2019;25:176-86.

7. Chowdhury S, Yadaiah N, Prakash C, Ramakrishna S, Dixit S, Gupta LR, et al. Laser powder bed fusion: a state-of-the-art review of the technology, materials, properties & defects, and numerical modelling. Journal of Materials Research and Technology. 2022;20:2109-72.

8. Gong H, Rafi K, Gu H, Starr T, Stucker B. Analysis of defect generation in Ti–6Al–4V parts made using powder bed fusion additive manufacturing processes. Additive Manufacturing. 2014;1-4:87-98.

This page contains no comments

9. Kasperovich G, Haubrich J, Gussone J, Requena G. Correlation between porosity and processing parameters in TiAl6V4 produced by selective laser melting. Materials & Design. 2016;105:160-70.

10. Yang J, Yu H, Yin J, Gao M, Wang Z, Zeng X. Formation and control of martensite in Ti-6Al-4V alloy produced by selective laser melting. Materials & Design. 2016;108:308-18.

11. Attar H, Calin M, Zhang LC, Scudino S, Eckert J. Manufacture by selective laser melting and mechanical behavior of commercially pure titanium. Materials Science and Engineering: A. 2014;593:170-7.

12. Meng LX, Yang HJ, Ben DD, Ji HB, Lian DL, Ren DC, et al. Effects of defects and microstructures on tensile properties of selective laser melted Ti6Al4V alloys fabricated in the optimal process zone. Materials Science and Engineering: A. 2022;830:142294.

13. Gong H, Rafi K, Gu H, Janaki Ram GD, Starr T, Stucker B. Influence of defects on mechanical properties of Ti–6Al–4V components produced by selective laser melting and electron beam melting. Materials & Design. 2015;86:545-54.

14. Greitemeier D, Dalle Donne C, Syassen F, Eufinger J, Melz T. Effect of surface roughness on fatigue performance of additive manufactured Ti–6Al–4V. Materials Science and Technology. 2016;32(7):629-34.

15. Hassanin H, El-Sayed M, Ahmadein M, Alsaleh N, Ataya S, Ahmed M, et al. Optimising Surface Roughness and Density in Titanium Fabrication via Laser Powder Bed Fusion. Micromachines. 2023:1642.

16. Mumtaz K, Hopkinson N. Top surface and side roughness of Inconel 625 parts processed using selective laser melting. Rapid Prototyping Journal. 2009;15(2):96-103.

17. Molinari A, Ancellotti S, Fontanari V, Iacob E, Luchin V, Zappini G, et al. Effect of Process Parameters on the Surface Microgeometry of a Ti6Al4V Alloy Manufactured by Laser Powder Bed Fusion: 3D vs. 2D Characterization. Metals. 2022;12(1):106.

This page contains no comments

18. Wang D, Lv J, Wei X, Lu D, Chen C. Study on Surface Roughness Improvement of Selective Laser Melted Ti6Al4V Alloy. Crystals. 2023;13(2):306.

19. Sun D, Gu D, Lin K, Ma J, Chen W, Huang J, et al. Selective laser melting of titanium parts: Influence of laser process parameters on macro- and microstructures and tensile property. Powder Technology. 2019;342:371-9.

20. Buhairi MA, Foudzi FM, Jamhari FI, Sulong AB, Radzuan NAM, Muhamad N, et al. Review on volumetric energy density: influence on morphology and mechanical properties of Ti6Al4V manufactured via laser powder bed fusion. Progress in Additive Manufacturing. 2023;8(2):265-83.

21. Song B, Dong S, Zhang B, Liao H, Coddet C. Effects of processing parameters on microstructure and mechanical property of selective laser melted Ti6Al4V. Materials & Design. 2012;35:120-5.

22. Promoppatum P, Yao S-C. Analytical evaluation of defect generation for selective laser melting of metals. The International Journal of Advanced Manufacturing Technology. 2019;103.

23. Dietrich K, Diller J, Dubiez-Le Goff S, Bauer D, Forêt P, Witt G. The influence of oxygen on the chemical composition and mechanical properties of Ti-6Al-4V during laser powder bed fusion (L-PBF). Additive Manufacturing. 2020;32:100980.

24. Promoppatum P, Srinivasan R, Quek SS, Msolli S, Shukla S, Johan NS, et al. Quantification and prediction of lack-of-fusion porosity in the high porosity regime during laser powder bed fusion of Ti-6Al-4V. Journal of Materials Processing Technology. 2022;300:117426.

25. Spierings A, Schneider M, Eggenberger R. Comparison of Density Measurement Techniques for Additive Manufactured Metallic Parts. Rapid Prototyping Journal. 2011;17:380-6.

This page contains no comments

26. Gong H, Rafi H, Nadimpalli K, Starr T, Stucker B. Defect Morphology in Ti-6Al-4V Parts Fabricated by Selective Laser Melting and Electron Beam Melting2013.

27. Wang Z, Xiao Z, Tse Y, Huang C, Zhang W. Optimization of processing parameters and establishment of a relationship between microstructure and mechanical properties of SLM titanium alloy. Optics & Laser Technology. 2019;112:159-67.

28. Carter LN, Villapún VM, Grover L, Cox SC. Exploring the duality of powder adhesion and underlying surface roughness in laser powder bed fusion processed Ti-6Al-4V. Journal of Manufacturing Processes. 2022;81:14-26.

29. Zhao X-l, Li S, Zhang M, Liu Y-d, Sercombe T, Wang S, et al. Comparison of the microstructures and mechanical properties of Ti 6AŒ 4V fabricated by selective laser melting and electron beam melting. Materials & Design. 2016;95:21-31.

30. Simson D, Subbu SK. Effect of Process Parameters on Surface Integrity of LPBF Ti6Al4V. Procedia CIRP. 2022;108:716-21.

31. du Plessis A, Yadroitsava I, Yadroitsev I. Effects of defects on mechanical properties in metal additive manufacturing: A review focusing on X-ray tomography insights. Materials & Design. 2020;187:108385.

32. Hojjatzadeh SMH, Parab ND, Guo Q, Qu M, Xiong L, Zhao C, et al. Direct observation of pore formation mechanisms during LPBF additive manufacturing process and high energy density laser welding. International Journal of Machine Tools and Manufacture. 2020;153:103555.

33. Pal S, Lojen G, Kokol V, Drstvensek I. Evolution of metallurgical properties of Ti-6Al-4V alloy fabricated in different energy densities in the Selective Laser Melting technique. Journal of Manufacturing Processes. 2018;35:538-46.

This page contains no comments

34. Qiu C, Adkins N, Attallah M. Microstructure and tensile properties of selectively laser-melted and of HIPed laser-melted Ti-6Al-4V. Materials Science and Engineering A. 2013;578:230-9.

35. Wei W-H, Shen J. Effect of laser energy density on microstructures and mechanical properties of selective laser melted Ti-6Al-4V alloy. International Journal of Materials Research. 2018;109.

36. Promoppatum P, Chayasombat B, Nyein Soe A, Sombatmai A, Sato Y, Suga T, et al. In-situ modification of thermal, microstructural, and mechanical responses by altering scan lengths in laser powder bed fusion additive manufacturing of Ti-6Al-4V. Optics & Laser Technology. 2023;164:109525.

37. du Plessis A. Effects of process parameters on porosity in laser powder bed fusion revealed by X-ray tomography. Additive Manufacturing. 2019;30:100871.

38. Martin AA, Calta NP, Khairallah SA, Wang J, Depond PJ, Fong AY, et al. Dynamics of pore formation during laser powder bed fusion additive manufacturing. Nature Communications. 2019;10(1):1987.

39. Khairallah SA, Anderson AT, Rubenchik A, King WE. Laser powder-bed fusion additive manufacturing: Physics of complex melt flow and formation mechanisms of pores, spatter, and denudation zones. Acta Materialia. 2016;108:36-45.

40. Hu YN, Wu SC, Withers PJ, Zhang J, Bao HYX, Fu YN, et al. The effect of manufacturing defects on the fatigue life of selective laser melted Ti-6Al-4V structures. Materials & Design. 2020;192:108708.

41. Mower TM, Long MJ. Mechanical behavior of additive manufactured, powder-bed laser-fused materials. Materials Science and Engineering: A. 2016;651:198-213.

This page contains no comments

42. Shunmugavel M, Polishetty A, Littlefair G. Microstructure and Mechanical Properties of Wrought and Additive Manufactured Ti-6Al-4V Cylindrical Bars. Procedia Technology. 2015;20:231-6.

43. Yadroitsev I, Krakhmalev P, Yadroitsava I, Du Plessis A. Qualification of Ti6Al4V ELI Alloy Produced by Laser Powder Bed Fusion for Biomedical Applications. JOM. 2018;70(3):372-7.

44. Frazier WE. Metal Additive Manufacturing: A Review. Journal of Materials Engineering and Performance. 2014;23(6):1917-28.

45. Zhao R, Chen C, Wang W, Cao T, Shuai S, Xu S, et al. On the role of volumetric energy density in the microstructure and mechanical properties of laser powder bed fusion Ti-6Al-4V alloy. Additive Manufacturing. 2022;51:102605.

46. Rafi HK, Karthik NV, Gong H, Starr TL, Stucker BE. Microstructures and Mechanical Properties of Ti6Al4V Parts Fabricated by Selective Laser Melting and Electron Beam Melting. Journal of Materials Engineering and Performance. 2013;22(12):3872-83.

47. He B, Wu W, Zhang L, Lu L, Yang Q, Long Q, et al. Microstructural characteristic and mechanical property of Ti6Al4V alloy fabricated by selective laser melting. Vacuum. 2018;150:79-83.

This page contains no comments

Figure captions

Fig 1. (a) 3D reconstructed surface topography of all samples from the software, (b) Mean surface roughness of the top surface, and (c) Mean surface roughness of the side surface.

Fig 2. Optical micrographs of as-built samples at various laser powers. (The same images were also used to calculate void fraction for the OM method.**)**

Fig 3. Cuboid of 3D geometry reconstructed by stitching 2D images from Micro-CT analysis. Higher magnification of void morphologies was also included.

Fig 4. (a) Comparison of void fraction among Archimedes' method, OM, and Micro-CT approach, (b) Comparison of void size distribution against various laser energy inputs, (c) Comparison of area void fraction along the sample thickness, and (d) Top view of a 3D reconstructed domain from micro-CT at the middle region.

Fig 5. (a) Engineering stress and strain curve, (b) Yield stress (σ_y), (c) Ultimate tensile stress (σ_{UTS}), (d) Young's modulus (E), and (e) Strain at break (ε)Fig 5. (a) Engineering stress and strain curve, (b) Yield stress (σ_y), (c) Ultimate tensile stress (σ_{UTS}), (d) Young's modulus (E), and (e) Strain at break (ε)

Fig 6. Correlation between tensile properties and the void fraction from Archimedes' method. (a) Yield stress (σ_y), (b) Ultimate tensile stress (σ_{UTS}), (c) Young's modulus (E), and (d) Strain at break (ε)

Fig 7. Scanning electron microscopy (SEM) images of as-built samples.

Fig 8. Fracture surface of as-built tensile samples from 75 W, 100 W, and 175 W groups. Higher magnification of specific areas was selected to represent the fracture behavior.

This page contains no comments

Click here to access/download;Figure;Fig 1.tif

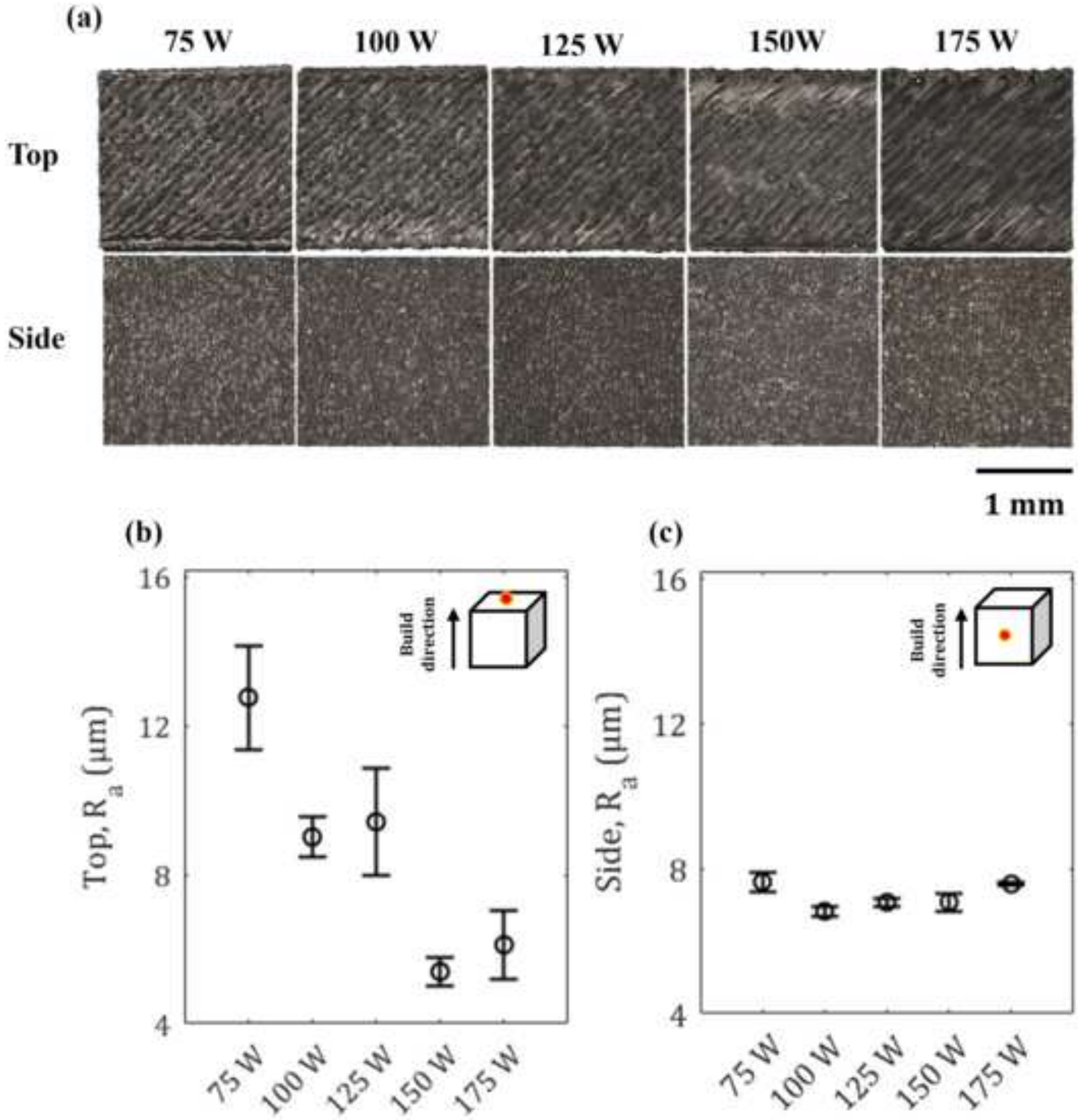

This page contains no comments

Fig2

Click here to access/download;Figure;Fig 2.tif

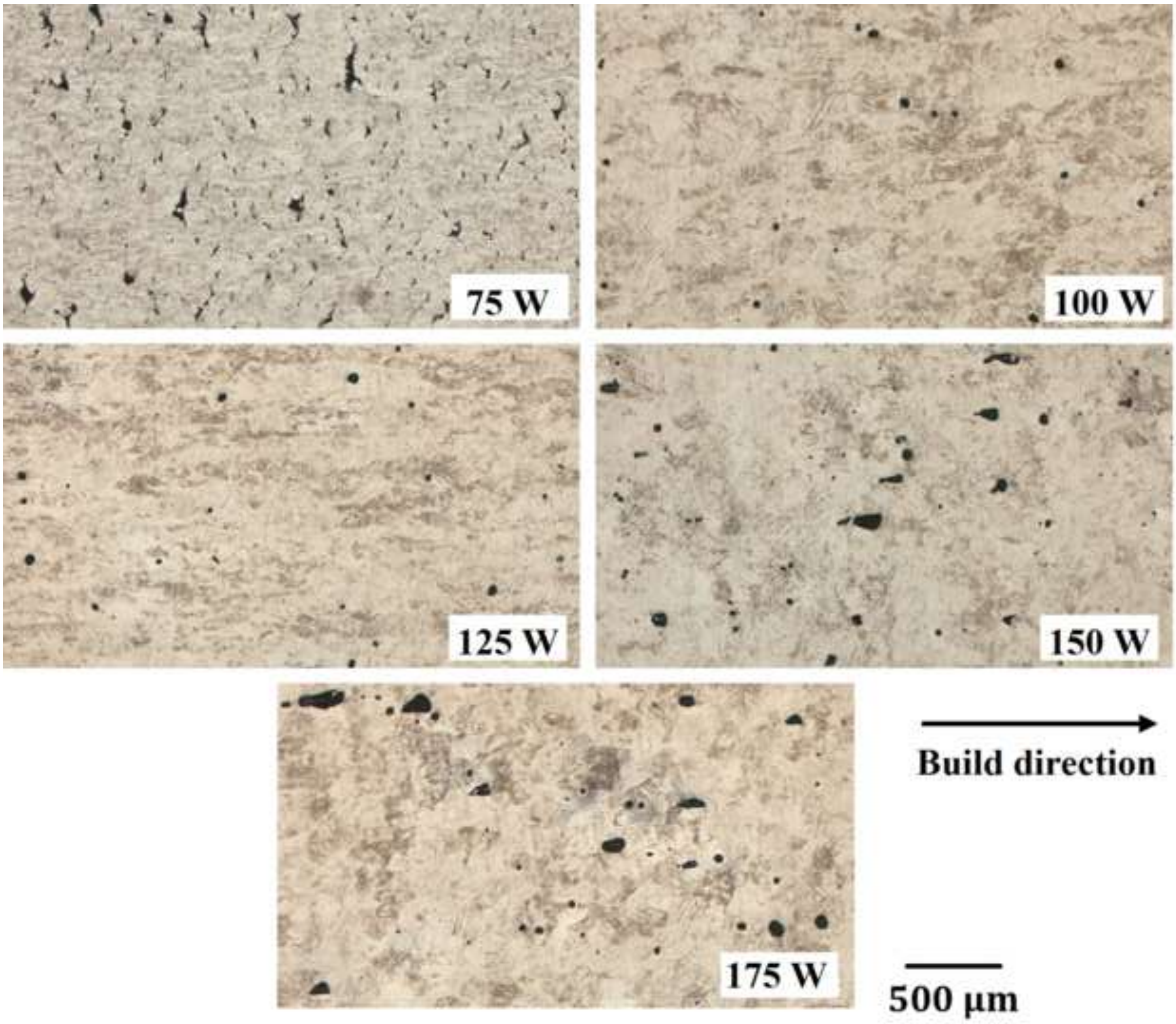

This page contains no comments

Fig3 Click here to access/download;Figure;Fig 3.tif

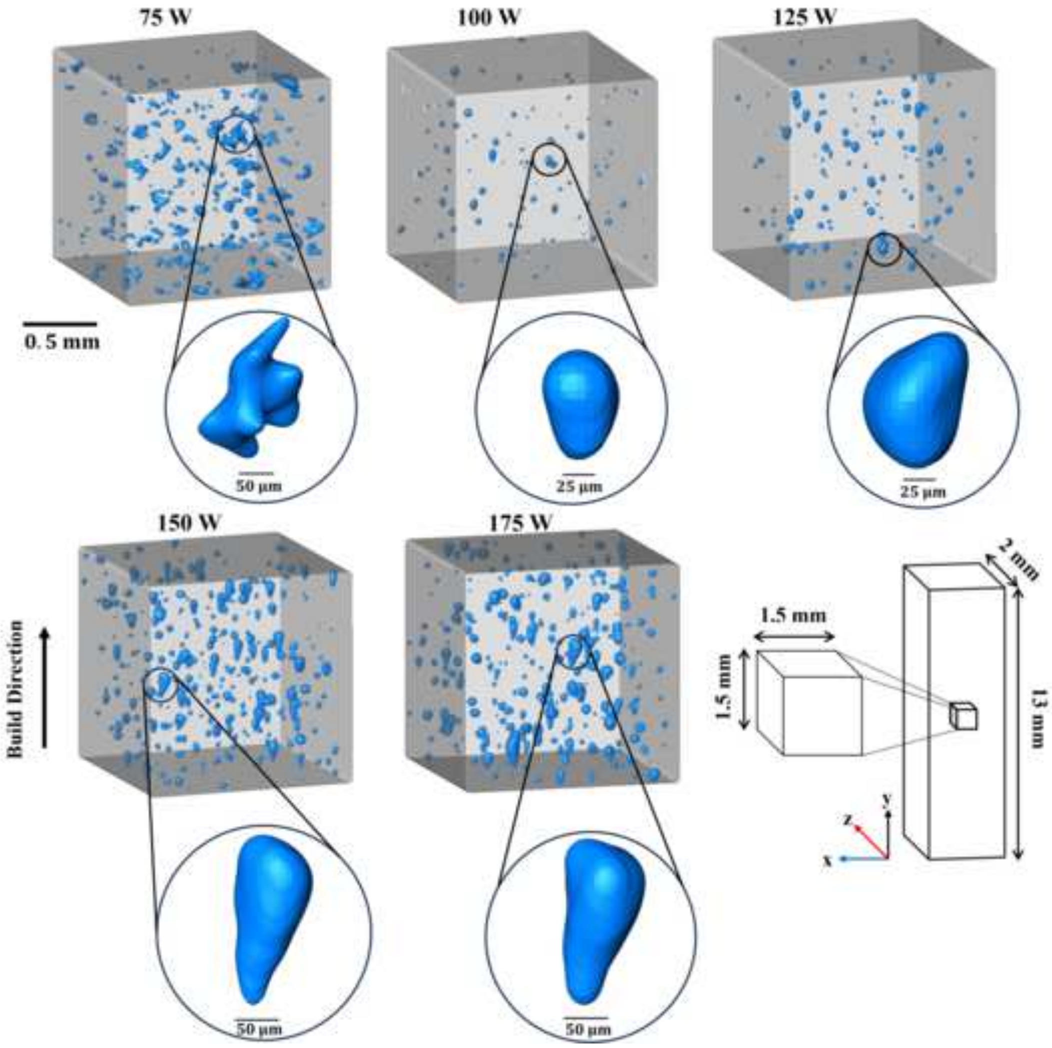

This page contains no comments

Fig4

Click here to access/download;Figure;Fig 4.tif

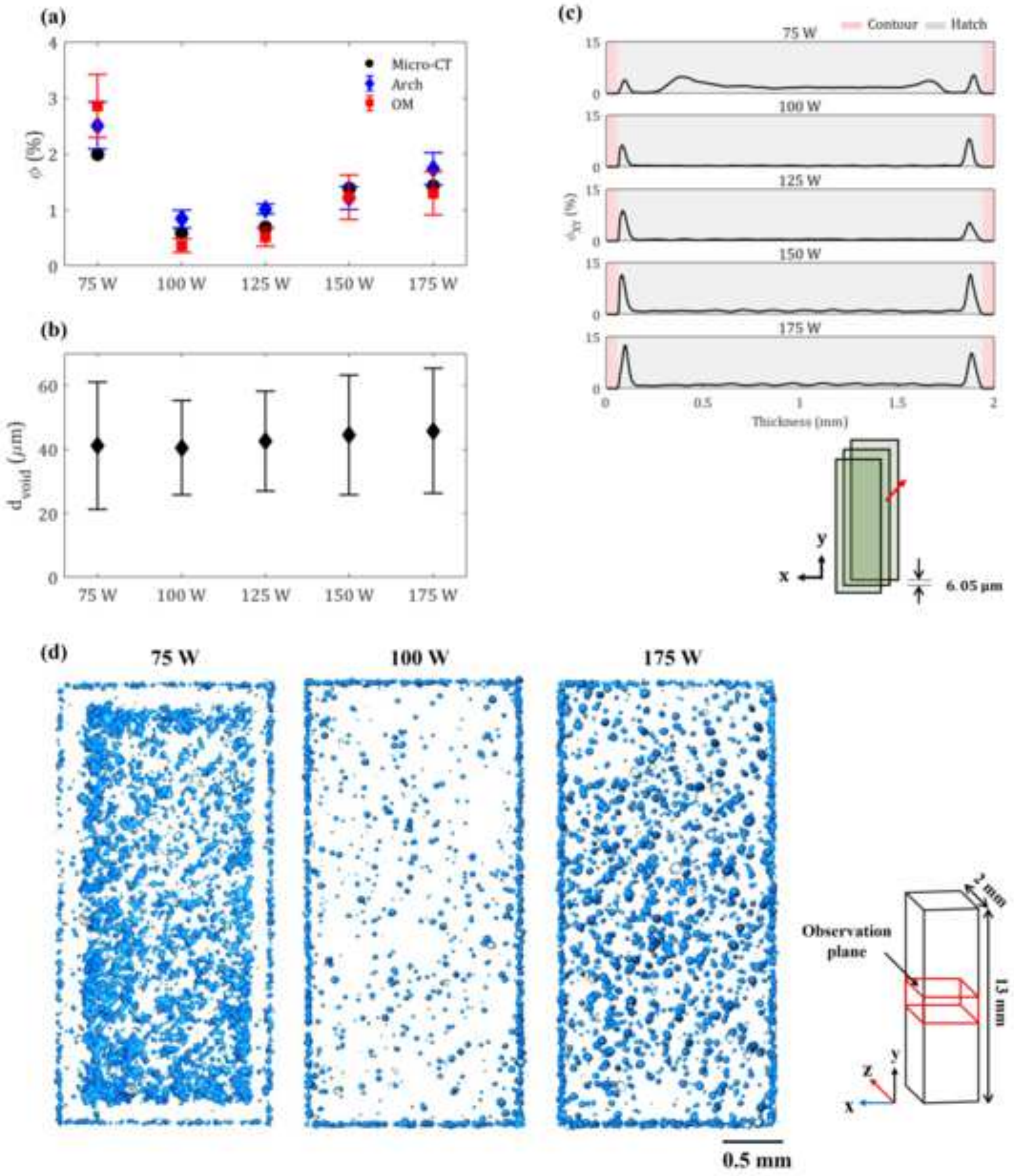

This page contains no comments

Click here to access/download;Figure;Fig 5.tif

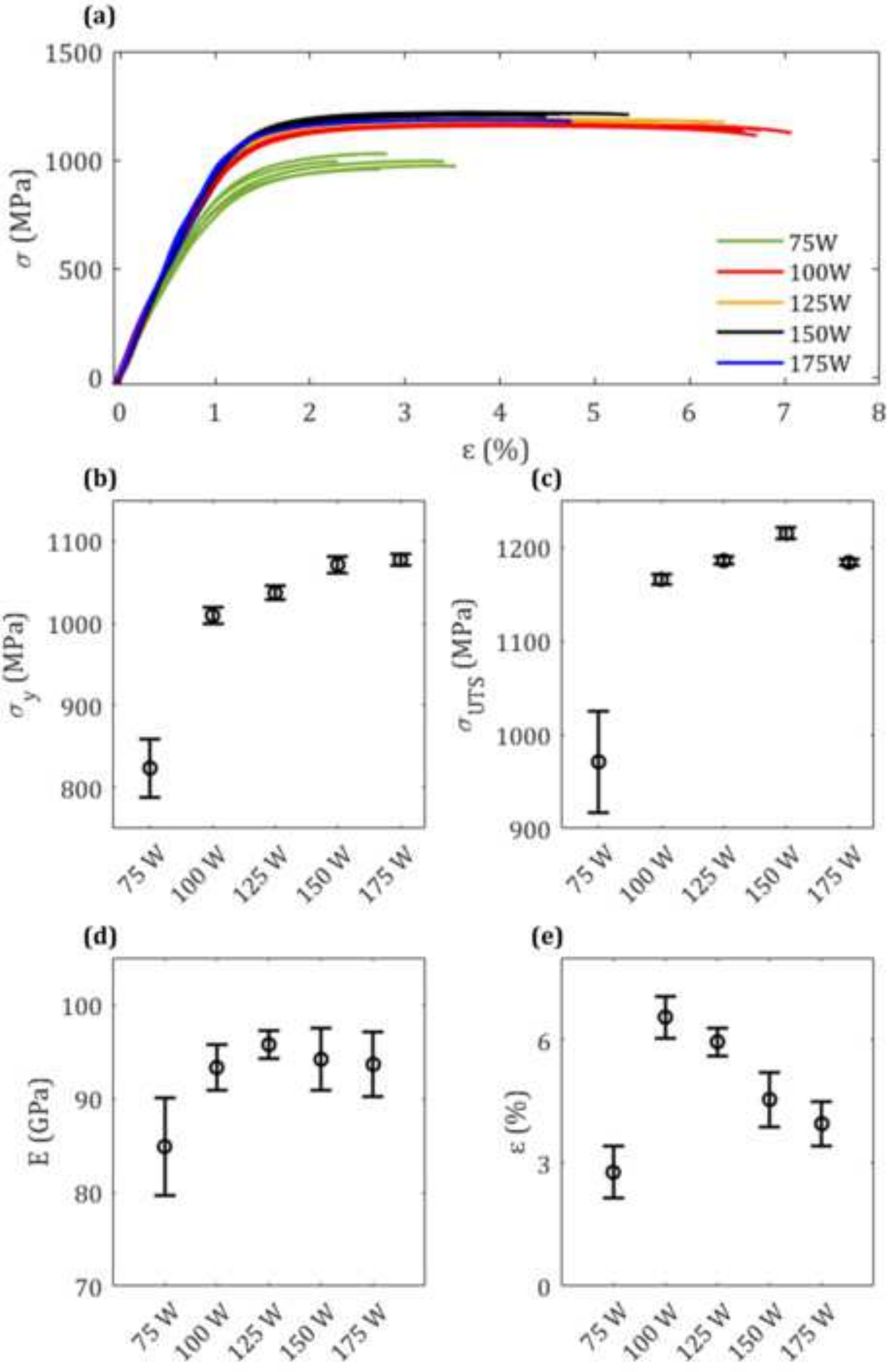

This page contains no comments

Fig6

Click here to access/download;Figure;Fig 6.tif

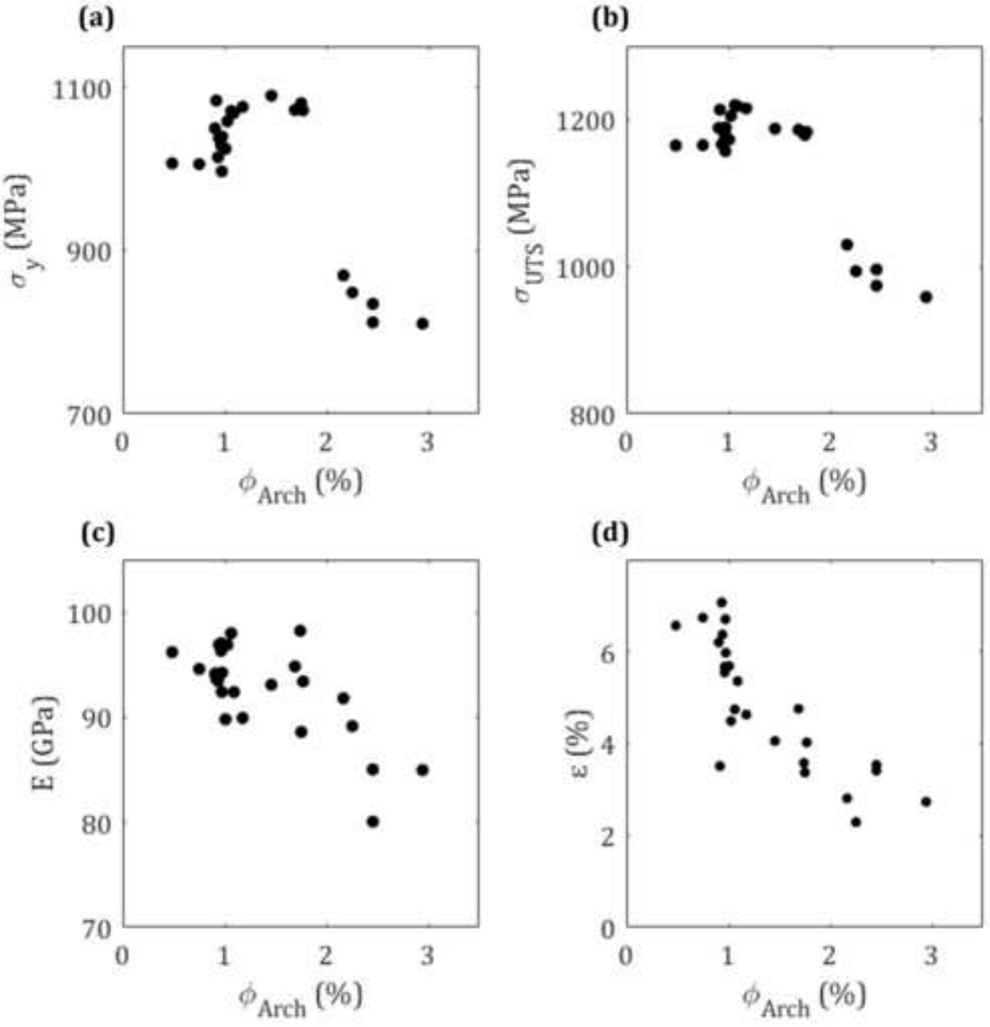

This page contains no comments

Click here to access/download;Figure;Fig 7.tif

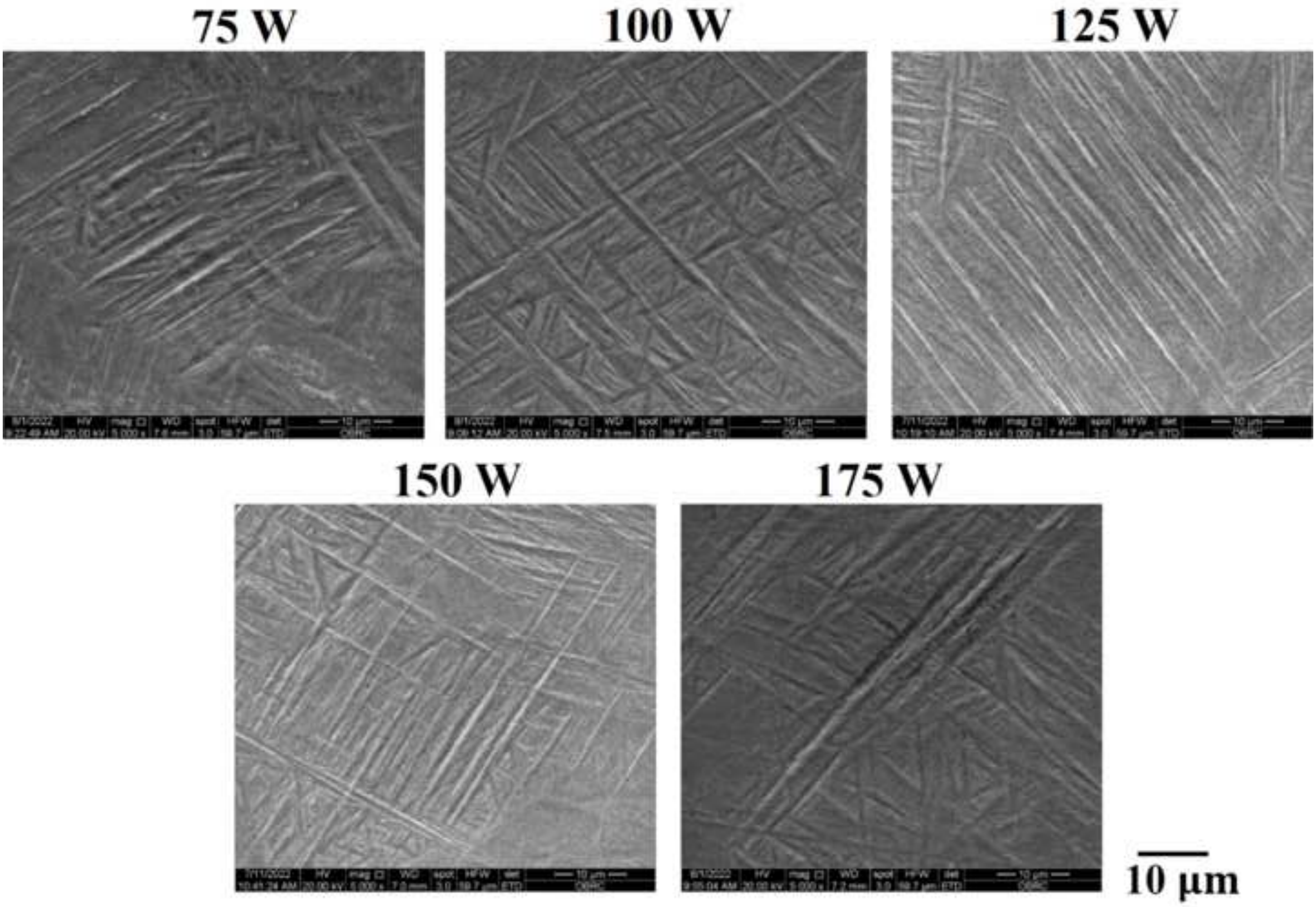

This page contains no comments

Fig8

Click here to access/download;Figure;Fig 8.tif

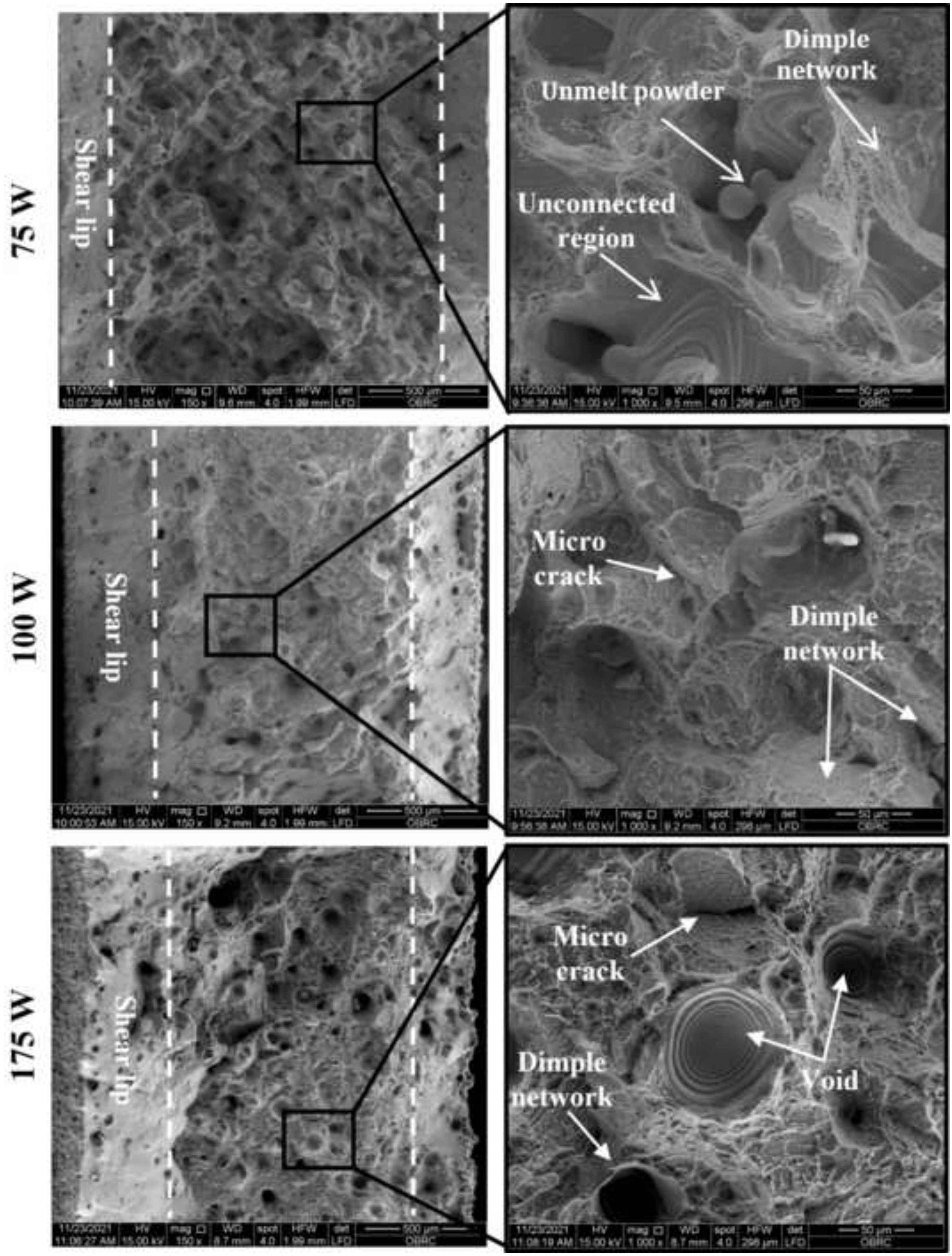

This page contains no comments
