# Supplementary material for: The critical role of optimized energy density in controlling void morphology and enhancing mechanical properties of L-PBF Ti-6Al-4V ELI alloy
Source: PLoS One. 2025 Jun 3;20(6):e0325276. doi: 10.1371/journal.pone.0325276 (PMC12133182; doi:10.1371/journal.pone.0325276)
Supplement: Attachment — Submitted filename: Response to Reviewers (PlosOne).docx [file pone.0325276.s003.docx]

**Responses to the reviewers’ comments**

The authors would like to thank the reviewers for their valuable comments. We have carefully considered each of the reviewers’ comments as shown in black and include our reply in blue. Please note that all the changes we have made in the revised version of the manuscript are highlighted with a yellow background and the reference numbers used in this reply refer to the bibliography in the revised manuscript.

**Responses to the Reviewer # 1’s comments**

Paper ‘The critical role of optimized energy density in controlling void morphology and enhancing mechanical properties of L-PBF Ti-6Al-4V ELI alloy’ presents an interesting topic related to optimal processing parameters and laser additive manufacturing. The influence of laser power on defects, microstructure and mechanical behavior of L-PBF fabricated TC4 alloy was studied. There's been a lot of researchers on this field, and some questions should be added to improve the quality of the manuscript.

1. The parameters that determine the density of laser energy are laser power and scanning speed, hatch spacing and layer thickness. There is no clear explanation for why only the laser power is indicated as a variable in Introduction. Also, the authors did not explain why the laser scanning speed of 1200 mm/s, layer thickness of 20 µm, hatch spacing of 110 µm, laser spot size of 30 µm a were selected in section of Materials and Methods.

**Response:** We would like to thank the reviewer for the comment. The choices of parameters used in our study are based on our pilot study and recommendation from 3D printer manufacturer. The choices of laser scanning speed, layer thickness, hatch spacing, and spot size were the default settings as recommended by machine manufacturer. To reflect the comment from the reviewer, we have added details in the revised manuscript.

**Page 6 Line 141-145**

2. The laser spot 30 µm was used in this paper, and it is not the same as the commonly used fiber laser with spot size of 70-100 µm. The authors should indicate the type and wavelength of the laser used in section of Materials and sample preparation.

**Response:** Thank you for your valuable comment. The laser fiber that this printer is this machine is Ytterbium (Yb) fiber laser with a wavelength 1,070 nm and spot size of 30 µm. Generally, the machine that utilizes the smaller spot size (30-50 µm), so-called high-resolution machines, are supposed to be used with medical and dental part which require finer details.

**Reference:**

Nagarajan, B.; Hu, Z.; Song, X.; Zhai, W.; Wei, J. Development of Micro Selective Laser Melting: The State of the Art and Future Perspectives. *Engineering* **2019**, *5*, 702–720.

We added more explanation about laser used in the Materials and Methods part.

The laser for the printer is Ytterbium (Yb) fibre laser with wavelength 1,070 nm and spot size of 30 µm [23].

**Reference:**

23. Pleass, C., & Jothi, S. (2018). Influence of powder characteristics and additive manufacturing process parameters on the microstructure and mechanical behaviour of Inconel 625 fabricated by Selective Laser Melting. *Additive Manufacturing*, *24*, 419-431.

**Page 6 Line 143-144**

3. The shape and size of tensile samples should give in section of Mechanical testing.

**Response:** Thank you for your valuable comment. We added more details about tensile samples in materials and methods.

The sizes of tensile sample followed ASTM E8, in which the detailed dimension could be also found in our previous work of Soe et al. [24 ].

**Reference:**

24. Soe, A. N., Sombatmai, A., Promoppatum, P., Srimaneepong, V., Trachoo, V., & Pandee, P. (2024). Effect of post-processing treatments on surface roughness and mechanical properties of laser powder bed fusion of Ti–6Al–4V. *Journal of Materials Research and Technology*, *32*, 3788-3803.

**Page 6 Line 144-145**

4. The oxygen, nitrogen, and hydrogen contents obviously increase with the increase of laser power. The author should explain the cause of this phenomenon.

**Response:** As recommended by the reviewer, the discussion on the increase of oxygen, nitrogen, and hydrogen contents was added in the revised manuscript.

The increased O, N, and H contents could be due to the higher melting pool temperature by higher laser power. This could make more oxygen or nitrogen trapped during melting and solidification because titanium is known for having a strong affinity for oxygen, nitrogen, and hydrogen especially in high temperatures. Thus, this might make the melt pool susceptible to contamination with a small amount of oxygen, nitrogen, and hydrogen that existed, although the chamber environment was controlled by argon. Furthermore, surface oxidation from heat generated by high laser power causes titanium oxide on the powder surface and in the melt pool. Therefore, these oxides can be entrapped in the solidified material, causing elevated oxygen levels [25].

**Reference:**

25. Dietrich, Kai & Diller, Johannes & Dubiez, Sophie & Bauer, Dominik & Forêt, Pierre & Witt, Gerd. (2019). The influence of oxygen on the chemical composition and mechanical properties of Ti-6Al-4V during laser powder bed fusion (L-PBF). Additive Manufacturing. 32. 100980. 10.1016/j.addma.2019.100980.

**Page 13 Line 295-303**

5. In Void defect analysis, the authors only analyzed the variation trend of laser power on the morphology and size of defects. It is also important for authors to provide a specific value for the size change. Furthermore, I believe that the described defect characteristics based on CT examination are not sufficient in this paper, and that the author should provide a more detailed description of defect characteristics and the correlation between these defects and process parameters.

**Response:** As suggested by the reviewer, we have provided a more detailed description of defect characteristics and the correlation between these defects and process parameters in the revised manuscript.

In addition, as shown in both Fig. 2 and Fig. 3, we observed the correlation between defect characteristics and process parameters. At a low power input of 75 W, the voids appear to have irregular shapes, indicating lack-of-fusion defects, which result from insufficient melt pool coverage on the metal powders. At laser powers of 100 W and 125 W, the number of voids decreases, and the voids become more spherical. This observation suggests the presence of gas pores, where trapped gas is released from the powders during the melting process. Furthermore, at high laser power inputs of 150 W and 175 W, the void shapes exhibit keyholing phenomena, characterized by voids elongated along the build direction, possibly influenced by recoil pressure during molten pool vaporization [7, 22].

**References:**

7. Chowdhury, S., Yadaiah, N., Prakash, C., Ramakrishna, S., Dixit, S., Gupta, L. R., & Buddhi, D. (2022). Laser powder bed fusion: a state-of-the-art review of the technology, materials, properties & defects, and numerical modelling. *Journal of Materials Research and Technology*, *20*, 2109-2172.

22. Promoppatum, P., & Yao, S. C. (2019). Analytical evaluation of defect generation for selective laser melting of metals. *The International Journal of Advanced Manufacturing Technology*, *103*, 1185-1198.

**Page 15 Line 330-338**

6. From Fig.5, the elongation of as-built samples is highest for the 100 W, and the tensile strength is highest for the 150 W. As stated in the article, the defects of the formed parts are at their lowest at 100W, however, the tensile strength is not the highest at this time. This indicates that the microstructure also has a great influence on the mechanical properties. It is necessary for the authors to explain the cause of mechanical properties change, combined with the change of microstructure as a result of laser power.

**Response:** As suggested by the reviewer, we have provided further discussion to highlight the competing effects between microstructures and defects on strengths and elongations.

Fig. 5 and Fig 6d shows that the elongation of as-built samples is the highest at a laser power of 100 W, while the tensile strength reaches its peak at a laser power of 150 W. Interestingly, the defects in the formed parts are minimized at 100 W, yet the strength is not maximized at this laser power. This observation suggests that defects and microstructures have competing influences on the mechanical properties. As discussed previously, the nature of defects transitions from lack-of-fusion to keyholing as laser power increases. Elongation is primarily determined by the extent of defects, as illustrated in Figure 6d. Conversely, tensile strength is influenced by additional factors, including microstructural features. Figure 7 highlights the acicular features of α' martensite. According to the previous work by Promoppatum et al. [32], the size of α' martensite significantly influences the strength of printed Ti-6Al-4V. Smaller α' martensite sizes result in higher yield strength due to the effect of grain strengthening. It is anticipated that higher laser power affects the cooling rates of solidified materials, thereby inducing variations in the features of α' martensite. Supporting this, Kaschel et al. [] reported that greater laser power produces smaller α' martensite structures. This finding aligns well with our experimental results, where the yield strength increases with higher laser power, as illustrated in Figure 5b. Overall, the mechanical properties of as-built samples are governed synergistically by defects and microstructural features, where defects primarily dictate elongation and microstructural characteristics, such as the size of α' martensite, play a critical role in determining tensile strength.

**References:**

32. Promoppatum, P., Chayasombat, B., Soe, A. N., Sombatmai, A., Sato, Y., Suga, T., & Tsukamoto, M. (2023). In-situ modification of thermal, microstructural, and mechanical responses by altering scan lengths in laser powder bed fusion additive manufacturing of Ti-6Al-4V. *Optics & Laser Technology*, *164*, 109525.

**Page 19-20 Line 439-457**

**Responses to the Reviewer # 2’s comments**

Thank you for your article. There are a number of inadequacies that will need to be addressed before this is at an acceptable quality level to be publishable.

1. The description of the manufacture of the samples needs significant improvement, detailing all the samples manufactured, their sizes, number off etc for each of the tests performed in their analysis.

**Response:** Thank you for your valuable suggestion. We have added more details about how to prepare samples in the Materials and Methods part.

**Page 6 Line 141-145**

2. The testing methods need to be more detailed to provide details of the sampling methods used to provide statistical robustness. Also, any stated value must have an associated error value stated.

**Response:** Thank you for your valuable comment. We have included SD values in the results.

3. The xCT software should be utilised in the analysis to extract useful information regarding the pores, including e.g. pore volume, pore size distributions, pore geometries (average roundness, average axis dimensions), etc. There is a lot of valuable data possible from xCT that you are not using.

**Response:** As suggested by the reviewer, we included further analysis on pore volume, pore size distributions, pore geometries (average roundness, average axis dimensions) in the revised manuscript.

**Page 10 Line 221-231**

4. There appears to be discrepancy between the results in Fig 3 and those in Fig 4. This needs to be explained.

**Response:** We would like to thank the reviewer for their comments. The reviewer pointed out a discrepancy between the results presented in Figures 3 and 4. Both figures display the 3D void shapes obtained from CT analysis, and we understand that the reviewer is referring to this as the perceived discrepancy. Figure 3 illustrates the void distribution within small extracted volume measuring 1.5 x 1.5 x 1.5 mm³. Additionally, Figure 4d also presents the void distribution; however, it provides a top-down view to highlight the void distribution within the x-z plane. As a result, although Figures 3 and 4d depict void distributions from the same samples, the differences in perspective and representation may give the impression of a discrepancy. To prevent the confusion, we have added in the revised manuscript the explanation.

Of note, although Figures 3 and Figure 4d depict void distributions from the same samples, the differences in perspective and representation may give the impression of a discrepancy. Figure 3 illustrates the void distribution within small extracted volume measuring 1.5 x 1.5 x 1.5 mm³ while Figure 4d provides a top-down view to highlight the void distribution within the XZ plane.

**Page 17-18 Line 393-396**

5. The discussion of the results is quite limited and needs improvement, with further analysis of the data required.

**Response:** As suggested by the reviewer, further discussion in the results and further analysis of the data are added in the revision.

6. Some conclusions and hypotheses are made that are unsubstantiated by the results as they stand and more detailed analysis of the results, and further sample analysis (advanced SEM techniques) will be required to be able to substantiate these.

**Response:** As suggested by the reviewer, we revised the conclusion and ensure that they are supported by the results carried out in the present work.

The present study conducted a comprehensive investigation to assess the impact of process parameters, particularly laser power, on the formation of void defects, surface quality, microstructural characteristics, and mechanical properties. The key findings from this study include:

- With laser power ranging from 75 to 175 W, elongation and yield strength varied approximately from 0.05–3% and 820–1080 MPa, respectively. Selecting optimal parameters are essential to minimize defects and maximize mechanical strength.
- As energy density increased, the top surface roughness of as-built samples decreased due to improved overlap of melting tracks. Roughness decreased from approximately 12 µm to 6 µm as laser power increased from 75 to 175 W. However, side surface roughness remained relatively unchanged, mainly consisting of partially adhered powder.
- Based on void shapes, variations in energy density led to three specific types of void defects: lack of fusion (LOF), gas pores (GP), and keyhole (KH) defects. The minimum void fraction was observed at a laser power of 100 W.
- All three methods—Archimedes’ method, optical microscopy (OM), and micro-computed tomography (Micro-CT)—exhibited a similar trend in void fraction. However, Micro-CT specifically revealed variations in area void fraction across the sample thickness and detected sub-surface pores.
- The mechanical properties of as-built samples are influenced by both defects and microstructural features. For instance, a substantial void fraction at 75 W laser power contributed to premature fracture. At higher laser power, yield strength increased due to α' grain refinement.

**Page 23-24 Line 524-546**

**Responses to comments in the manuscript**

**Page 6**

You need to provide a description of the manufacture of all the samples, i.e their geometries, numbers off, position on the bed relative to the recoater direction and gas flow direction (position on the bed can greatly influence quality due to weld spatter, obscuration of the laser by weld fumes, variation in bed density, variation in beam quality at extremes of bed etc).

How were the samples removed from the build plate?

**Response:** As suggested by the reviewer, ASTM standard used in this research was explained. The details related to the fabrication of the test coupons were already reported in our prior research. Additionally, the method used for removing the samples was also mentioned in the following paragraph.

**Page 6 Line 141-145 and Page 7 Line 159-161**

**Page 7**

How many samples were assessed? How many readings were made per sample? What sample area was used?

**Response:** For the surface roughness test, we utilized 3 samples from each laser power group, with measurement taken in 3 areas per sample. Each measurement area was 2x2 mm^2^, and 10X magnification was used for the investigation.

**Page 7 Line 172-173**

**Page 8**

What sample geometries, volumes, masses were used? How were the samples extracted from the L-PBF samples? How many samples were tested for each of the energy level groups?

**Response:** We added more details about O/N/H analysis in Materials and Methods part. We measured one sample randomly chosen to represent O/N/H content of each energy group.

**Page 8 Line 184-187**

**Page 9**

What was sample geometry, size?

**Response:** We measured the density of the tensile sample which the size follows ASTM E8.

Is that 5 different specimens for each energy group or one sample measured 5 times?

**Response:** We measured 5 samples for each laser power group. Details are included in the main text.

**Page 9**

Need to have reference for all these values.

**Response:** We had added references for each value (Ref 28 and 29) in the manuscript.

**Page 9 Line 205-206**

Is that 5 sections taken from one sample or 5 samples, each with one cross-section? Sample size and geometry?

**Response:** One sample was randomly selected from each group and was sectioned into size 4x4 mm. The sample was hot mounted for OM analysis.

**Page 9 Line 207-208**

**Page 10**

This detail should be in the methodology section, including the methods used to extract the cubes.

**Response:** We had written the detail of method how to extract the cubes in the Materials and Method part.

This needs to be expanded on to determine the resolution of the system, e.g the minimum void diameter that can be resolved. What is sample geometry and volume? How many xCT taken of each energy level?

**Response:** The In-vitro Micro-Computed Tomography (Micro-CT) (Skyscan 1173, BRUKER company, Belgium) with a voxel size of 6.05 $\mu m$ was used to analyze the void defect in as-built samples. Rectangular specimens (4-mm width, 13-mm length, and 2-mm thickness) were taken from the gauge length of as-built samples.

The resolution of Micro-CT depends on the chosen voxel size, which limit the detection of voids smaller than three times voxel size ($\approx$ 18 $\mu m$).

So we added more explanation about Micro-CT analysis in the Materials and Methods part. **Page 10 Line 221-231**

**Page 11**

What was the sample geometry? Was this adhering to a standard, and if so, which one?

**Response:** The sample geometry was already mentioned in Materials and sample preparation section. We followed ASTM E8 standard. The same standard configuration has been used to publish several research articles from our research group.

**Page 13**

Could also be due to a higher melt pool temperature and lower melt viscosity that affects the melt pool shape and therefore the surface quality. This should be researched and discussed in reference to the literature.

**Response:** Thank you for your valuable comment. We agree and had added more discussion here.

However, a slight increase in surface roughness was observed in 175 W, which could be attributed to several factors. First, the elevated laser power generates melt pool instabilities, leading to the formation of keyholing and spattering. These instabilities result in the formation of surface irregularities, thereby increasing roughness [39, 40]. Secondly, the higher melt pool temperature can also cause more powder to adhere to the vertical side of the samples and further elevated surface roughness [36].

**Page 12-13 Line 282-287**

**Page 13 Table 1**

These values need an error value after each one. You say that you measured 5 for each sample so your error should be: standard deviation / sqareroot

**Response:** We only measured one sample per group and compared the results with manufacturing data and ASTM. So this is a reason that Table 1 doesn’t show the SD (error).

**Page 15**

You should use the xCT software to extract useful information regarding the pores, including e.g pore size distributions, pore geometries (average roundness, average axis dimensions), etc. There is a lot of valuable data possible from xCT that you are not using.

**Response:** We have already included the data on pore size distribution, pore geometries, pore fraction, and average pore diameters, which also give insights into the roundness of the pore morphology, as displayed in Fig 3 and Fig 4.

This detail should be in the methodology section, including the methods used to extract the cubes.

**Response:** The details of Micro-CT was already mentioned in Materials and Method, with our prior research cited as references. In this paragraph, the description was included to explicitly explain Fig. 3.

**Page 16**

What does this mean? Why do the images in Figure 4 not represent what is seen in Figure 3? Why is the density of pores so much higher in Figure 4? If a slice is taken near the centre of the images of those in Fig 3, the density of pores is much lower than shown in Fig 4 (unless Fig 4 is a cumulative image of many different layers?) Please explain this descrepancy, and provide more detail as to how Fig 4 is obtained.

**Response:** Fig. 4 d shows the top view of 3D volume with multiple slices, taken from the middle region of reconstructed 3D geometry, to indicate that the porosity is higher at the near surface region as it is evident in Fig. 4 b, which shows the pore fraction along the thickness. Furthermore, the illustration is already provided in Fig. 4d.

**Page 17**

What does this show? What are the black lines, are these error bars or the minimum and maximum levels? This is not clear. A better method to show this data is using box-plots:

**Response:** The black lines represent error bars, indicating the standard deviation of the void size distribution for each laser power group.

A useful metric would also be the circularity (or average axis lengths) as gas and lack of bonding pores are circular whilst KH pores are not, and this metric (from xCT) would be able to identify the distribution of each. Provide this information with accompanying data and discussion.

**Response:** As reviewer mentioned, it is quite obvious that the types of pores determine the circularity. Moreover, we have already discussed that three types of pores (LOF, GP, and KH) were found in the as-built samples depending on laser groups in Fig. 3. In that respect, LOF would have lower circularity compared to GP and KH. Whereas, it can also be realized that GP had a higher circularity against LOF and KH. Therefore, we did focus on the void size distribution in our research. By taking into account of void size distribution, we were able visualize the deviation of void size within each pore type.

**Page 17 Line 375-386**

**Page 17**

You need to provide an explanation for the increased internal void peaks (second subsurface). I would suggest that it is due to power ramping of the laser that drops the powder below that required for complete fusion, before it rises up to the 75W. You need to determine from the system what the power ramping sequence used is, and how long would you expect the power to reach 75W from 0W (and thus, at a scan speed of 1200 mm/s, does this relate to the general width of the 2nd peaks)?

Alternatively, it could also be due to the scanner acceleration being low, so that the laser is at 75W whilst the scan speed is still well below 1200mm/s, increasing the energy density to values above which vaporisation and KH voids form.

You will need to investigate the shape of the pores in this peak to see if their morphology aligns with lack of fusion or key hole porosity.

Please include this and make the necessary discussion. Without this level of detail the discussion and results are fairly inconsequential as you have not identified the cause.

**Response:** We would like to thank the reviewer for pointing out very interesting observation. As suggested, we have discussed the potential reason for the increased internal void peaks in the revised manuscript. We had added more discussion as following:

As seen from Fig. 4c, the 75 W group exhibits a unique void distribution pattern, characterized by high-intensity peaks not only at the subsurface but also at a distance of 0.2 mm from the primary peaks on both sides. The appearance of these secondary peaks of internal voids can be attributed to several possible factors. One explanation is the laser power ramping during the deposition process. During the power ramp-up phase, the laser power may fall below the threshold required for complete fusion, resulting in void formation. Alternatively, the distinct void distribution could be influenced by the thermal accumulation effect caused by the scan pattern. For instance, at the onset of laser deposition, the surrounding powder particles and the solid substrate are likely to be at a temperature close to the chamber temperature. However, as the laser continues to deposit material, it raises the temperature of the surrounding region. Consequently, the powder in subsequent laser passes may melt more effectively, leading to a non-uniform void distribution.

**Page 17 Line 375-386**

**Page 18**

Why use this measurement of density? State why you are using this and not the other methods used.

**Response:** There are three methods that we use for measuring void fraction, which are Archimedes method, optical micrographs, and micro-CT. We used three methods for verifying the result, and the result showed the same trend. According to Spiering et al.[15], the Archimedes method showed higher accuracy and repeatability values than other methods, therefore, Archimedes was utilized in this part.

I would like to see the points for each of the five energy levels displayed differently (different colours or different icons) on each graph so that the link between the energy level density and mechanical property can be seen easily in one graph (Fig.6)

**Response:** We improved the Fig 6 using different colors following the reviewer’s suggestion.

**Page 19**

You need to provide a specific reference to each of the points raised, e.g. one for defects, one for microstructure and one for stress.

**Response:** Thank you for your comments, we discussed more and cited references for each point in the paragraph.

**Page 19 Line 432-435**

**Page 21**

This statement is not justified since you have no data on α' grain size refinement for these samples. You will need to provide microstructural analysis, with grain size distributions for the α' martensite grains to be able to state this. Please do so.

The images in Fig 7 do are not suitable for this analysis and do not show anything more than a typical Ti64 microstructure. They do not add value to the argument or discussion.

**Response:** Thank you for your valuable comment. We added more images of microstructural analysis showing the grain size distribution and α' martensite (new figure is added - **Fig 7**.) to explain the result. We also reorder the figure numbers.

**Page 21**

You need to extend the analysis of this microstructure to show whether or not any α' Martensite grain refinement is occurring.

**Response:** We analyzed and discussed more in this part.

The microstructure of as-built samples exhibits a fully α' martensitic microstructure for all laser power, as exhibited in Fig 7. In the current study, columnar prior $\beta$ grains found along the building direction in Fig 7 commonly occur in L-PBF due to the repeated cycle of the heating and remelting process [42]. With increasing laser power at a constant scanning speed of 1200 mm/sec, the width of columnar β grains tends to increase. For 75 W, $\beta$ grains are narrower, as shown in Fig 7a. When higher laser power was applied, the width of the prior grain size increased in Fig 7b and c. However, 150 W and 175 W caused the transition from columnar to equiaxed morphology of prior $\beta$ grains in Fig 7d and e. This characteristic is related to the excessive energy density that causes unstable melt pool and distorts the grain as reported by Han et al.[55].

**Page 21 Line 471-480**

**Page 23-24**

This conclusion cannot yet be substantiated by the results.

**Response:** We have rewritten the conclusion to be substantiated by the present results.

**Page 23-24 Line 524-546**
