# Supplementary material for: The critical role of optimized energy density in controlling void morphology and enhancing mechanical properties of L-PBF Ti-6Al-4V ELI alloy
Source: PLoS One. 2025 Jun 3;20(6):e0325276. doi: 10.1371/journal.pone.0325276 (PMC12133182; doi:10.1371/journal.pone.0325276)
Supplement: Attachment — Submitted filename: Response to Reviewers (PlosOne) second revision.docx [file pone.0325276.s004.docx]

**Responses to the reviewers’ comments**

The authors would like to thank the reviewers’ valuable comments. We have considered each of the reviewers’ comments as shown in black and include our reply in blue. Please note that all the changes we have made in the revised version of the manuscript are highlighted with a yellow background and the reference numbers used in this reply refer to the bibliography in the revised manuscript.

**Responses to the Reviewer # 2’s comments**

1. Errors are now provided with results, but they need to be presented to the appropriate level of precision. E.g. P28, 12.772±1.394 um, should be written as 13±1, 7.631±0.265 should be written as 7.6±0.3 etc. Please amend this throughout.

We would like to thank the reviewer for the comment. We changed the decimal point as recommended by the reviewer.

Page 12 Line 262:

At 75 W, the top surface roughness is approximately 12.8 ± 1.4 μm which is statistically significant than other groups.

Furthermore, there is no statistically significant difference in surface roughness between all laser groups, apart from 75 W and 175 W, which have slightly higher mean roughness values of 7.6 ± 0.3 and 7.6 ± 0.1 $\mu m$, respectively.

2. Table 1 P29 still requires an assessment of error in the wt% values.

For Table 1, as recommended, we analyzed and displayed the error number from three tests.

Page 13 Line 303:

Table 1. The chemical composition from O/N/H analysis (wt.%)

| Remark | O | N | H |
| --- | --- | --- | --- |
| Ti-6Al-4V powder | 0.1012 ± 0.0077 | 0.0234 ± 0.0015 | 0.0033 ± 0.0003 |
| 75 W | 0.1132 ± 0.0010 | 0.0274 ± 0.0007 | 0.0034 ± 0.0005 |
| 100 W | 0.1124 ± 0.0037 | 0.0316 ± 0.0050 | 0.0032 ± 0.0004 |
| 125 W | 0.1294 ± 0.0040 | 0.0308 ± 0.0028 | 0.0034 ± 0.0002 |
| 150 W | 0.1189 ± 0.0035 | 0.0298 ± 0.0021 | 0.0031 ± 0.0003 |
| 175 W | 0.1229 ± 0.0116 | 0.0322 ± 0.0086 | 0.0047 ± 0.0003 |
| ASTM F2924-14 | 0.2 (Max) | 0.05 (Max) | 0.015(Max) |

3. P32 No assessment of error in the dimensions of the voids. All these dimensions require an error value. Without that you cannot assess if the changes between samples is statistically significant.

We sincerely appreciate the reviewer's comments. Based on our understanding, the assessment of errors in void dimensions was already included in the manuscript. However, it may not have been stated clearly, potentially leading to ambiguity. Therefore, we have revisited and clarified the assessment of errors in void dimensions herein.

The dimensions of the voids were measured using CT data, with a sampling volume of 1.5 × 1.5 × 1.5 mm³. For samples processed at laser powers of 75, 100, 125, 150, and 175 W, the numbers of voids captured within the control volumes was 1392, 294, 322, 636 and 524, respectively. Based on these captured voids, we determined the average void size and the standard deviation of void dimensions, with the results plotted in Figure 4b.

In the revised manuscript, we have also included Table 2 providing additional details on the analysis of void sizes. The changes made in the revision are outlined below.

Page 16 Line 368:

In addition, the details of the analysis of void sizes and the assessment of dimensional variability of voids are shown in Table 2.

**Table 2. Details on the analysis of void sizes**

| Laser power | Numbers of voids under 1.5×1.5×1.5 mm³ sampling volume | Void size (µm) |
| --- | --- | --- |
| 75 | 1392 | 41.2±20.0 |
| 100 | 294 | 40.2 ±15.3 |
| 125 | 322 | 41.7±17.7 |
| 150 | 636 | 42.1±20.0 |
| 175 | 524 | 45.7±19.5 |

**Responses to the Reviewer # 4’s comments**

4. The abstract is perfect.

We would like to thank you for comment.

5. It is better to provide references for formulas and equations.

As recommended, we provided for formulas and equations used in the manuscript. Changes in the revision could be seen below.

Page 7 Line 152:

The volumetric energy density ($E_{v}$) is expressed as follows in Eq. (1) [26]:

Page 7 Line 172:

The average surface roughness was reported as mean profile roughness ($R_{a})$, which is the integral of the absolute value of the surface profile that deviates from the mean line for a specific length, as given by Eq. (2) [24].

Page 9 Line 196:

Following Archimedes’ method, as-built tensile samples were weighed in air and water to calculate void fraction (ф_Arch_) (Eq.(3)) using an analytical balance (METTLER TOLEDO, classic, AB104-S, Switzerland) [27, 28].

Page 9 Line 212:

A void fraction $(ф_{\mathrm{OM}})$ was calculated by dividing the area of black pixels by the total number of pixels in binary images (Eq. (4)) [27].

Page 10 Line 226:

The void fraction of Micro-CT (ф_Micro-CT_) is determined from the volume of void voxels divided by the total voxel of 3D reconstructed domains (Eq.(5)) [27].

6. Increase the quality of Figure 1.

We improved the quality of Fig 1 and attached it in the submission of revised manuscript.
