# Supplementary material for: The critical role of optimized energy density in controlling void morphology and enhancing mechanical properties of L-PBF Ti-6Al-4V ELI alloy
Source: PLoS One. 2025 Jun 3;20(6):e0325276. doi: 10.1371/journal.pone.0325276 (PMC12133182; doi:10.1371/journal.pone.0325276)
Supplement: Attachment — Submitted filename: Response to Reviewers (PlosOne) third revision.docx [file pone.0325276.s005.docx]

**Responses to the reviewers’ comments**

The authors would like to thank the reviewers’ valuable comment. Please note that all the changes we have made in the revised version of the manuscript are highlighted with a yellow background.

**Responses to the Reviewer # 2’s comment**

Errors are now provided with results, but they need to be presented to the appropriate level of precision. E.g. P28, 12.772±1.394 um, should be written as 13±1, 7.631±0.265 should be written as 7.6±0.3 etc". Throughout the manuscript, uncertainties should be quoted to 1 significant figure and associated values quoted to this same precision.

We would like to thank the reviewer for the comment. We corrected the quotation of values and uncertainties as recommended by the reviewer except Table 1: Chemical composition from ONH analysis because the data has 4 decimal places which cannot be changed.

Page 12 Line 262:

At 75 W, the top surface roughness is approximately 13 ± 1 μm

Page 12 Line 267:

which have slightly higher mean roughness values of 7.6 ± 0.3 and 7.6 ± 0.1 $\mu m$, respectively.

Page 16 Line 362:

At 75 W, the mean void diameter is approximately 41 ± 20 $\mu m$, indicating considerable variability due to insufficient energy for stable melting. Similarly, at 175 W, the mean void diameter increases to 46 ± 20 $\mu m$,

Page 17 Line 372:

| Laser power | Numbers of voids under 1.5×1.5×1.5 mm³ sampling volume | Void size (µm) |
| --- | --- | --- |
| 75 | 1392 | 41 ± 20 |
| 100 | 294 | 40 ± 15 |
| 125 | 322 | 42 ± 18 |
| 150 | 636 | 42 ± 20 |
| 175 | 524 | 46 ± 20 |
